# Liver epigenome changes in patients with hepatopulmonary syndrome: A pilot study

**DOI:** 10.1371/journal.pone.0245046

**Published:** 2021-02-25

**Authors:** Nuria Mendoza, Eva Rivas, Roberto Rodriguez-Roisin, Tamara Garcia, Miquel Bruguera, Alvar Agusti, Rosa Faner

**Affiliations:** 1 Centro de Investigación Biomédica en Red de Enfermedades Respiratorias (CIBERES), Madrid, Spain; 2 Institut d’Investigacions Biomèdiques August Pi i Sunyer (IDIBAPS), Barcelona, Spain; 3 Department of Anesthesia, Hospital Clinic, Barcelona, Spain; 4 Respiratory Institute, Hospital Clinic, Barcelona, Spain; 5 University of Barcelona, Barcelona, Spain; Chuo University, JAPAN

## Abstract

The hepatopulmonary syndrome (HPS) is defined by the presence of pulmonary gas exchange abnormalities due to intrapulmonary vascular dilatations in patients with chronic liver disease. Changes in DNA methylation reflect the genomic variation. Since liver transplant (LT) reverts HPS we hypothesized that it may be associated with specific liver epigenetic changes. Thus, the aim of this study was to investigate the role of the liver epigenome in patients with HPS. We extracted DNA from paraffin embedded liver tissue samples from 10 patients with HPS and 10 age-, sex- and MELD (Model for End-stage Liver Disease)-matched controls. DNA methylation was determined using the 850K array (Illumina). Weighted Gene Co-expression Network Analysis (WGCNA) was used to identify modules related to defining physiologic characteristics of HPS. Only 12 out of the 20 liver biopsies (7 HPS and 5 controls) had sufficient quality to be analyzed. None of the 802,688 DNA probes analyzed in the case control comparison achieved a significant False Discovery Rate (FDR). WGCNA identified 5 co-methylated gene-modules associated to HPS markers, mainly related to nervous and neuroendocrine system, apoptotic processes, gut bacterial translocation, angiogenesis and vascular remodeling ontologies. To conclude, HPS is associated with nervous/neuroendocrine system and vascular remodeling related liver epigenetic changes.

## Introduction

The hepatopulmonary syndrome (HPS) is defined by the presence of an arterial oxygenation defect caused by intrapulmonary vascular dilatations in patients with chronic liver disease, mostly liver cirrhosis (LC) [1–5]. The prevalence of the HPS in patients assessed for liver transplant (LT) ranges between 5–30% [6–8]. Thirty years ago, we were the first to show that patients with LC [9–11] exhibit reduced or abolished pulmonary vascular tone, also distinctive of HPS. More recent studies have proposed a number of putative biological mechanisms [12–17], but the pathobiology of HPS still remains elusive to date. The progression of chronic liver damage to cirrhosis and HPS is highly variable between patients and the course disease is often clinically unpredictable [18]. It is well established that there are several non-genetic factors that influence the progression of other chronic liver diseases, including diet, exercise, microbiome and alcohol consumption [15]. These non-genetic factors have been associated with the modulation of organ functionality through changes in the epigenome [18–20]. DNA methylation is one of the main epigenetic mechanisms that regulates gene transcription. It involves the addition of a methyl group to a cytosine base adjacent to a guanine base (CpG site) in regulatory regions of DNA, and it is associated with decreased gene expression [21]. We hypothesized that HPS could be related to specific epigenetic liver changes, as recently described in non-alcoholic fatty liver disease [22], obesity and diabetes [23]. Accordingly, this study seeks to: *(1)* compare the liver epigenome of patients undergoing LT, with and without HPS; and, *(2)* relate epigenome differences to the defining pathophysiological markers of HPS.

## Material and methods

The ethics committee of the Hospital Clinic de Barcelona reviewed and approved the study (HCB/2014/1045) and written consent was required.

### Study design and ethics

This is a controlled study performed in paraffin embedded liver biopsies obtained from patients with LC (with and without HPS) who required LT from 1996 to 2014 in our hospital. Samples were stored in the Biobank of our institution. Although the number of samples is limited, they are representative of the population of patients with LC with and without HPS as all the patients requiring LT in our hospital during 18 years have been included. For this study patients were recruited thought the revision of medical records of our hospital. All patients signed their informed consent form before LT. We confirm that all methods were carried out in accordance with relevant guidelines and regulations, and all experimental protocols were approved by the ethics committee of the Hospital Clinic (HCB/2014/1045).

### Patients

We identified 10 liver biopsies from transplanted LC patients with moderate-to-severe HPS (PaO_2_ < 70 mmHg) [2, 3, 5] and 10 biopsies from transplanted LC patients with normal lung function and no HPS, matched for sex, age and severity of liver disease (MELD) [24]. Patients with liver carcinoma and/or primary biliary cirrhosis were excluded.

### Epigenetic analysis

#### DNA extraction

Total DNA was extracted from paraffin preserved liver samples using the RecoverAll™ Total Nucleic Acid Isolation Kit for FFPE (Ambion, Life Technologies, US) following manufacturer instructions. DNA quantity was determined by Nanodrop (Thermo Scientific, Willmington, US) and quality by Qubit (Life Technologies, US). Before running the arrays, the suitability of DNA for methylation analysis was assessed using FFPE QC kit (Illumina, San Diego, US), as recommended by Illumina. Bisulfite conversion was performed with the EZ-96 DNA Methylation Kit (Zymo research, CA, US) following manufacturer instructions. The Infinium MethylationEPIC Beadchip [25] (Illumina), which covers over 850,000 CpG sites of the human genome, was used to assess the methylation levels at Hospital de la Fe, Valencia, Spain.

#### DNA methylation analysis

The Illumina EPIC Array was used to determine the DNA methylation status of >800.000 CpG sites in liver samples, and the *minfi* package [26] was used for quality check, normalization and processing of raw data. The *minfi* package also allowed us to identify methylated probes located in autosomal chromosomes, excluding those located in single nucleotide polymorphisms (SNP) positions. The Limma package (R platform) was then used on the filtered matrix of M values to calculate the differential methylation adjusting for age and sex in the linear model. CpGs were assigned to the nearest gene, so that probes with no gene were excluded from further analysis. Likewise, the R package GeneAnswers with the L5 of GeneOntolgy [27] was used to identify functional enrichment in the CpGs differentially methylated in patients with and without HPS; significant enrichment was defined as a False Discovery Rate (FDR) <0.05. Finally, REVIGO [28] was used to evaluate term overlap and to obtain a visual summary of functional enrichment results. Data is available from the GEO repository database (GSE162984).

#### Weighted Gene Co-expression Network Analysis (WGCNA)

To identify modules of co-methylated genes related to functional and clinical characteristics, the probes with nominal p value <0.05 in the case/control analysis were used to build the co-methylation network using the WGCNA R package [27, 29]. To do so, probes were collapsed to genes. In the modules of interest, functional enrichment was calculated with GeneAnswers [27], considering significant enrichment as FDR <0.05.

### Statistical analysis

Results are presented as number (proportion) or median [interquartile range]. Continuous and categorical variables were compared between patients and controls using Mann-Whitney test and Fisher’s Exact test, respectively. All statistics were computed with R.

## Results

### Patient characteristics

Although we originally included in the study liver biopsies from 10 patients with and 10 without HPS, only 12 of them (7 with and 5 without HPS) passed the *minfi* quality control of the 850K (EPIC) methylation array results (S1 Table in S1 File) and could therefore be included in the analysis. Table 1 compares the demographic and main clinical characteristics of these 12 patients. There were no significant differences between groups with respect to age, sex, etiology or severity of cirrhosis. Alcohol abuse and hepatitis C virus infection were the most frequent causes of LC. Spirometry was within normal limits in both groups but, by design, pulmonary gas exchange was severely impaired in patients with HPS but within the normal range in those without HPS (AaPO_2_, 60±13 vs 8±5 mmHg; PaO_2_, 54±6 *vs* 102±10 mmHg; DL_CO_, 54±19 *vs* 80±10% predicted, respectively; p<0.05 each). Liver biopsies stained with hematoxylin-eosin were blindly reviewed by an expert liver pathologist (MB). Neither the type of LC, inflammatory activity, presence of vascular proliferation within septa, sinusoidal dilatation, necrotic areas, cholangiolar proliferation, steatosis or cholestasis were different between patients with and without HPS (S2 Table in S1 File).

**Table 1:** Demographic and clinical characteristics of patients with LC without and with HPS (mean ±SD).

	Without HPS	With HPS	p values
	(n = 5)	(n = 7)	
**Age, years old**	53±7	52±9	NS
**Sex (female/male)**	2/3	3/4	NS
**Body mass index, kg/m**^**2**^	26±3	26±5	NS
**Etiology**	Viral = 2	Viral = 3	NS
	Alcohol = 2	Alcohol = 3	
	Viral+Alcohol = 1	Viral+Alcohol = 1	
**CHILD-PLUG classification**	A, 0	A, 3	NS
	B, 4	B, 2	
	C, 1	C, 2	
**MELD Score**	15±2	15±5	NS
**Smoker, n (packs-year)**	4 (12±9)	5 (28±19)	NS
**Forced Vital Capacity (FVC, % ref.)**	100±11	95±12	NS
**Forced Expiratory Volume in 1 sec. (FEV**_**1**_**, % ref)**	100±6	97±8	NS
**FEV**_**1**_**/FVC (%)**	0.79±0.06	0.81±0.07	NS
**DL**_**CO**_**, (% ref)**	80±10	54±19	**<0.05**
**AaPO**_**2**_**, mmHg**	8±5	60±13	**<0.01**
**PaO**_**2**_**, mmHg**	102±10	54±6	**<0.01**
**PaCO**_**2**_**, mmHg**	34±5	30±6	NS

MELD, Model for End-stage Liver Disease; FVC, forced vital capacity; FEV_1_, forced expiratory volume in 1 s; DL_CO_, single breath lung diffusing capacity for carbon monoxide; AaPO_2_, alveolar-arterial oxygen gradient; PaO_2_, partial pressure of arterial oxygen; PaO_2_, partial pressure of arterial carbon dioxide; NS, not significant.

### DNA methylation analysis

**Fig 1:**
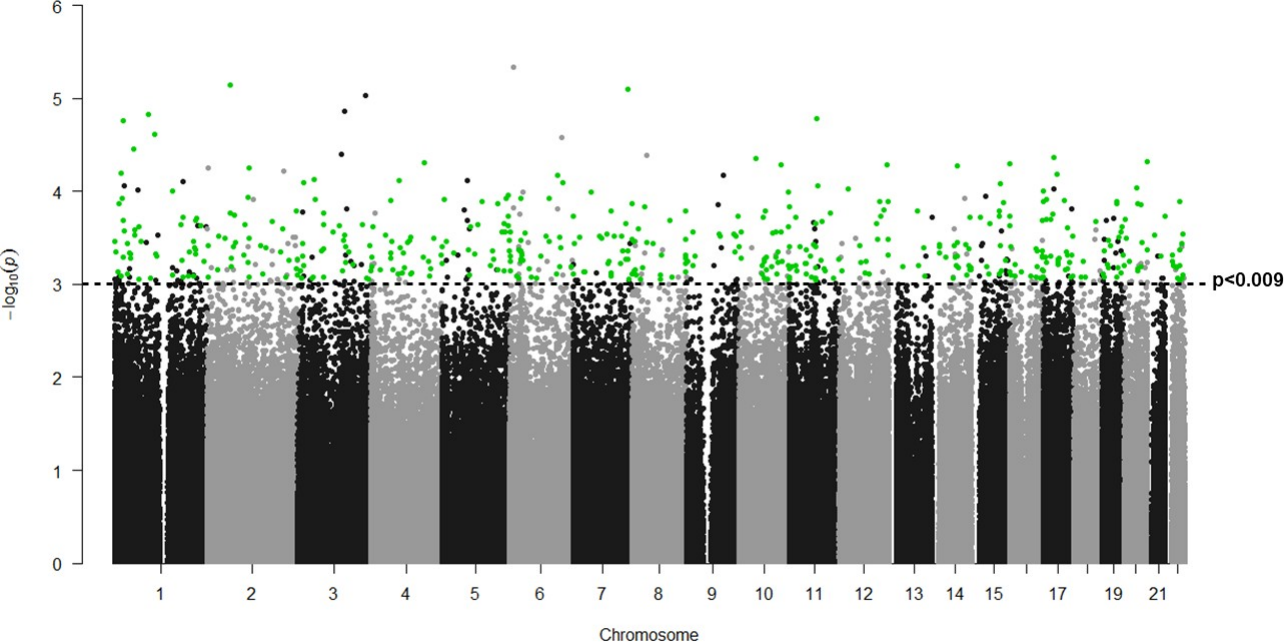
Manhattan plot of differentially methylated genes in LC with and without HPS. *P* values (-log10[p]) are plotted against their respective positions on each chromosome. Green dots indicate the 465 positions differentially methylated with p values <0.0009, FC>|0,4| and associated to a single gene. Any positions which do not follow these criteria are represented in black dots.

None of the 802,688 DNA probes contrasted between patients with and without HPS achieved a significant False Discovery Rate (FDR) lower than 0.05, likely in relation to the small sample size available (7 HPS vs. 5 non-HPS; n = 12). Yet, to explore potential differences between cases and controls, we decided to use less stringent statistical criteria (nominal p<0.05, not FDR corrected). Accordingly, our results have to be considered exploratory. By doing so, we identified 48,386 differentially methylated probes between patients with and without HPS (Fig 1).

S1 Table (in S1 File) provides the list of the probes with the lowest p values (p<0.0009) and highest absolute fold change (FC>|0.4|), which represented the most variable probes among study groups (n = 443). Table 2 lists the top differentially methylated genes with a positive (i.e., more methylated in controls) or negative (i.e., more methylated in patients with HPS) FC, ordered by their respective p values.

**Table 2:** Top differentially methylated genes with a positive or negative Fold Change (FC) ordered by |FC|.

Positive FC: higher methylation in patients without HPS				Negative FC: higher methylation in patients with HPS			
Gene	Probe ID	FC	p values	Gene	Probe ID	FC	p values
**ORMDL3**	cg09155575	1.21	6.47E-05	**4 GBP**	cg00273257	-1.16	1.50E-05
**VAV3**	cg19103456	0.99	2.44E-05	**HIVEP2**	cg05786041	-1.12	8.14E-05
**MYT1**	cg11839596	0.94	4.75E-05	**ANKRD28**	cg22424370	-1.12	8.14E-05
**PAX8**	cg23156509	0.91	5.57E-05	**FAM168A**	cg16322681	-1.10	1.63E-05
**CNTNAP2**	cg16508202	0.89	7.93E-06	**SCAMP3**	cg24034289	-1.05	9.95E-05
**CPNE1**	cg15917394	0.87	9.20E-05	**SPEN**	cg08892464	-1.03	6.32E-05
**TBC1D2B**	cg07690703	0.80	8.34E-05	**VTI1A**	cg00592484	-1.01	5.20E-05
**C10orf142**	cg19100384	0.79	4.44E-05	**XPO1**	cg26131881	-0.97	7.15E-06
**IL15**	cg25690285	0.76	4.88E-05	**RCHY1**	cg13431998	-0.96	7.63E-05
**CRAMP1L**	cg15231222	0.75	5.09E-05	**TMEM132D**	cg22386460	-0.87	5.15E-05
**GUCY2EP**	cg16855265	0.72	8.74E-05	**DCAF5**	cg16121759	-0.84	5.34E-05
**MYO18A**	cg02376256	0.70	4.31E-05	**TCEA3**	cg18167160	-0.78	1.72E-05
**ANKRD11**	cg04200026	0.66	9.90E-05	**KRAS**	cg18249781	-0.77	9.35E-05
**TTC39A**	cg24797045	0.64	3.46E-05	**ANO10**	cg19647107	-0.76	7.41E-05
				**LAMA2**	cg10671433	-0.63	6.68E-05

**Fig 2:**
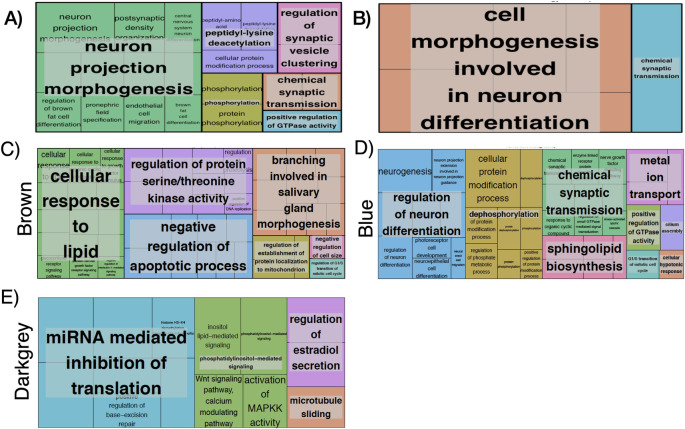
Visual summary of GO terms (Treemap in REVIGO(46)) for: (A) 443 genes total list p<0.0009, (B) Negative FC (FC >0.4, FDR <0.05), (C) Brown WGCNA module, (D) Blue WGCNA module, (E) Darkgrey WGCNA module. Each box is a single ontology cluster, which are joined into super-clusters of loosely related terms, shown by the same color and named with a representative term. Larger boxes mean more significant enrichment. Letter C denotes control patients (without HPS).

To identify biological processes associated with these 443 most differently methylated probes, we computed the gene ontology (GO) and KEGG pathway enrichment analysis using the GeneAnswers R package. We identified 44 functionally GO enriched terms with a FDR<0.05 (S3 Table in S1 File, summarized in Fig 2A) which were related to neuron projection morphogenesis, regulation of synaptic vesicle clustering, peptidyl-lysine deacetylation, chemical synaptic transmission, phosphorylation and positive regulation of GTPase activity (Fig 2A).

We then computed the Gene Ontology (GO) enrichment including only those genes with a positive FC (250) or those with a negative FC (188). This analysis identified three GO enriched terms in the former and 17 in the later (FDR <0.05). The 3 ontologies associated with a positive FC were related to pepdidyl-lysine deacetylation, histone H3 deacetylation and positive regulation of hydrolase activity (S4 Table in S1 File). The 17 ontologies associated with a negative FC were related to cell morphogenesis involved in two main processes: neuron differentiation, chemical synaptic transmission and trans-synaptic signaling (S4 Table in S1 File, summarized in Fig 2B).

Finally, we investigated if any of the genes listed in Table 2 (i.e., those with higher magnitude of change) were included in any of the gene ontologies identified for positive and negative FC, separately (S3 and S4 Tables in S1 File), and found that no genes were included in the three ontologies more methylated in patients non-HPS patients (positive FC). However, two genes (LAMA2 and KRAS) were included in 7 of the 18 ontologies found to be more methylated in HPS patients (i.e., negative FC). These 7 ontologies were associated to cell morphogenesis involved in neuron differentiation, axonogenesis, neuron projection morphogenesis, gliogenesis and plasma membrane bounded cell projection morphogenesis (S4 Table in S1 File).

### Weighted Gene Co-expression Network Analysis (WGCNA)

To identify groups of co-methylated genes related to clinical characteristics of interest, we performed WGCNA analysis with probes having a nominal p value <0.05. WGCNA identified 18 modules (labeled by a color name), each containing a minimum of 30 co-methylated genes (S1A Fig).

**Fig 3:**
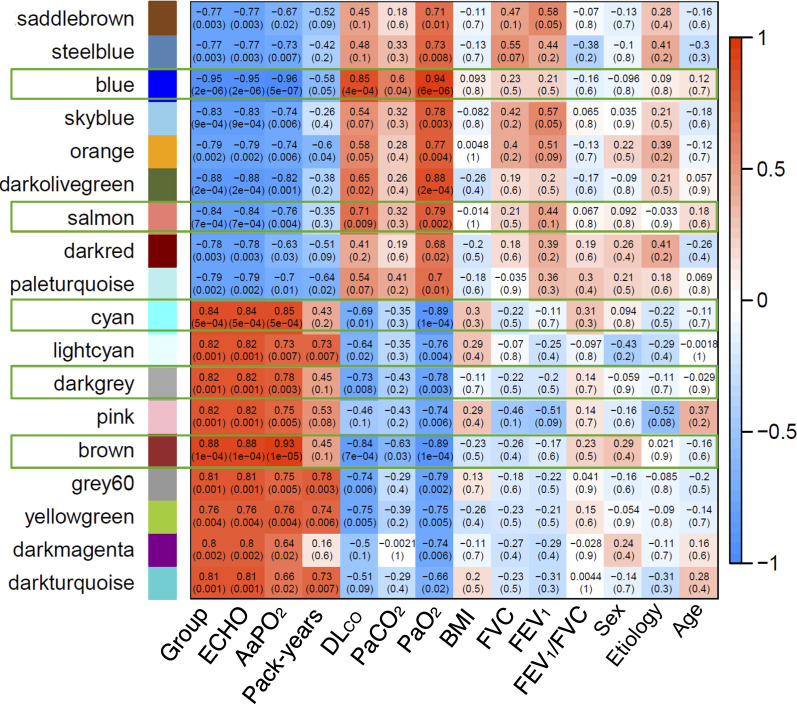
Heatmap of DNA methylation data based on unsupervised hierarchical clustering that shows the correlation between the modules identified by WCGNA and a number of clinically relevant variables. Red and blue cells indicate a positive or negative correlation coefficient (see scale). Only framed modules were further analyzed. Abbreviations: *group*: patients with or without HPS; *ECHO*, contrast-enhanced echocardiography; *AaPO*_*2*_, alveolar-arterial oxygen gradient (mmHg); cumulative smoking exposure (pack-years); *PaCO*_*2*_, partial pressure of arterial carbon dioxide (mmHg); *DLco*, single-breath diffusing capacity for carbon monoxide (% reference); *PaO*_*2*_, partial pressure of arterial oxygen (mmHg); *BMI*, body mass index (kg/m^2^); FVC, forced vital capacity (% reference); *FEV*_*1*_, forced expiratory volume in the 1^st^ second (% reference).

Fig 3 illustrates a heat-map representing the potential relationship (positive [red], none [white] or negative [blue]) between these 18 modules of co-methylated genes and a number of relevant clinical characteristics (bottom axis). Differences were very clear between patients with and without HPS (so-called “groups”). No module was related to sex, body mass index, etiology of LC or spirometric variables (FEV_1_, FVC or FEV_1_/FVC). By contrast, five modules (28%; *blue* [8947 genes], *brown* [3037 genes], *cyan* [128 genes], *darkgrey* [64 genes] and *salmon* [131 genes]) were differentially associated with the four pulmonary gas exchange descriptors that best characterize HPS (high AaPO_2_, low PaO_2_, reduced DLco and positive contrast-enhanced echocardiography [ECHO]) (Fig 3). Three out of these 5 modules (*brown*, *blue* and *darkgrey*) presented relevant biological processes and molecular function ontologies enriched with a FDR <0.05 (S4 Table in S1 File, summarized in Fig 2). The *brown module* (S5 Table in S1 File, summarized in Fig 2C) included ontologies related to cellular response to lipids, salivary gland morphogenesis, regulation of serine/threonine kinase activity, negative regulation of apoptotic process, regulation of establishment of protein localization to mitochondrion, negative regulation of cell size. The later included phosphorylation, epithelial cell differentiation and apoptotic process. *The blue* module (S5 Table in S1 File, summarized in Fig 2D) included ontologies related to regulation of neuron differentiation, dephosphorylation, chemical synaptic transmission, metal ion transport, positive regulation of GTPase activity, sphingolipid biosynthesis, cilium assembly, cellular hypotonic response. Interestingly, both the *brown* and *blue* modules included ontologies related to G1/S transition of mitotic cell cycle. Finally, the *darkgrey* module (Fig 2E) included ontologies related to regulation of cellular macromolecule biosynthetic processes, phosphatidylinositol-mediated signaling, miRNA mediated inhibition of translation, regulation of estradiol secretion and microtubule sliding.

## Discussion

The results of this pilot show, for the first time to our knowledge, that the key defining characteristics of the HPS, namely pulmonary gas exchange abnormalities and presence of intrapulmonary vascular dilatations [2], are associated with specific liver epigenome changes related to nervous and neuroendocrine system, vascular remodeling and angiogenesis gene ontologies.

We were among the first to define the gas exchange pathophysiologic hallmarks of HPS primarily characterized by moderate-to-severe ventilation-perfusion imbalance, and reduced oxygen diffusion transfer to the alveoli in the presence of intrapulmonary vascular dilatations [17]. Subsequent studies proposed a number of putative biological mediators, including nitric oxide (NO) [12, 13, 17] and endothelin-1 (ET-1) [16], and identified a few genetic polymorphisms related to angiogenesis [15] and inflammation [14] in human and experimental HPS. To our knowledge, no previous study has explored potential liver epigenetic changes in patients with HPS. Likewise, no previous study has used WGCNA to get an integrated view of gene co-expression networks and to relate it with specific clinical and functional features of HPS. Although WGCNA does not provide causal information, it enables the identification of regulatory genes underlying various clinical phenotypes of interest [27, 29].

It is likely that due to the relatively small number of liver biopsies available for analysis (n = 12) in regards with the number of DNA probes analyzed (n = 802,688), no DNA probe achieved the minimum FDR level of statistical significance (Fig 1); hence, our findings herein should be considered exploratory. With this caveat in mind, however, some observations reported here may provide novel information of potential interest.

First, pulmonary angiogenesis and vascular remodeling are considered key pathogenic features of HPS [3, 30], mostly involving the activation of vascular endothelial growth factor (VEGF)-dependent pathways [14, 31–33]. In this context, it is of note that some of the top differentially methylated genes identified in patients with HPS (Table 2) were also related to angiogenesis, including VAV3 [34], PAX 8 [35], K-RAS [36] and LAMA2 [37].

Second, we also identified other significant ontologies associated with HPS, including neurological processes such as neurogenesis, neuron differentiation and gliogenesis. Interestingly, the role of the neuroendocrine and autonomic nervous system in the liver function has been well described, including glucose metabolism, regulation of blood flow and vasculature, hepatic regeneration and fibrosis [38]. Importantly, several neuropeptides and neurohormones, namely melatonin and A-calcitonin gene-related peptide (a-CGRP), have been shown to regulate angiogenesis, vasodilatation and inflammatory cytokines [39].

Finally, WGCNA identified five specific modules of co-methylated genes that were associated with key defining characteristics of HPS, i.e. increased AaPO_2_, decreased PaO_2_, low DL_CO_ and intrapulmonary vascular dilations by echocardiography (Fig 3).

A more detailed analysis identified a number of gene ontologies with FDR<0.05 of 3 of these 5 modules (Fig 2), including the regulation of cellular response to lipids, endothelium development and morphogenesis, regulation of apoptotic process, regulation of cell size/cycle, regulation of methylation and translation, and regulation of estradiol secretion. All of them are potentially relevant for a better understanding of the pathobiology of HPS. First, the regulation of the cellular response to lipids relates to the response to bacterial lipoproteins which, in turn, may relate to gut bacterial translocation, a potentially relevant pathogenic mechanism of HPS [40]. Second, epithelial cell differentiation/migration and endothelial development ontologies can be related to abnormal angiogenesis, pulmonary vascular dilatations, endothelial dysfunction and vascular remodeling [41], all key components of the vascular features of HPS. Third, the regulation of estradiol secretion includes ontologies related hormone secretion, thus neuroendocrine pathways. Interestingly, increased levels of progesterone and estradiol have been associated to intrapulmonary vascular dilatations and gas exchange abnormalities in patients with cirrhosis [42]. In fact, progesterone and estradiol levels are increased in patients with HPS [43]. Likewise, it is of note that previous studies have related hypothalamic-pituitary-adrenal (HPA) axis dysfunction with LC, portal hypertension and HPS [44, 45]. The HPA axis regulates many liver functions through neuroendocrine forward signaling, such as glucogenesis, lipid metabolism, growth hormone signaling and inflammation [46]. Interestingly, these ontologies were also identified by WGCNA in the current patients with HPS. Finally, the interaction of proliferation and apoptosis of vascular endothelial cells is a pivotal mechanism of vascular remodeling [34], and we observed that apoptotic processes (GO:0006915) were related to DL_CO_ (a functional marker of abnormal pulmonary circulation). It is of note that Roberts *et al*. reported that CAV3 and TGFB1 (both included in this apoptotic ontology) were associated with HPS [15]. Collectively, therefore, these observations support our working hypothesis, namely that HPS is associated with distinct liver epigenome changes. This can also contribute to explain the well-established clinical observations that, on the one hand, not all patients with LC develop HPS, and on the other, that HPS is not associated with the severity of LC, as reflected by the MELD score [2, 47, 48]. Future research is needed to confirm our findings here and, eventually, to facilitate the identification of novel potential therapeutic targets for the HPS.

The main strength of our study is that it investigates a previously unexplored hypothesis using state of the art technology in a small but carefully selected identified subset of patients with LC and HPS undergoing LT. We acknowledge, however, that the small number of liver biopsies (n = 12) of sufficiently high quality available for DNA methylation analysis is a limitation of our study. Difficulties in recruiting larger numbers of patients with HPS transplanted in our hospital for this type of complex translational approaches are similar to those noted in previous studies [49–51]. In any case, this has unfortunately restricted our ability to reach the required FDR value associated with the use of more than 800,000 DNA probes and implies that our findings need to be considered hypothesis generating and require validation by other studies. Likewise, we did not have access to frozen liver samples (which may have allowed us to explore transcriptomic differences) or serum samples (to contrast potential systemic biomarkers between the two groups of patients studied). Nonetheless, we hope that our observations will encourage other researchers in the field to confirm and expand our findings herein.

## Conclusions

This pilot study shows that the functional and structural defining characteristics of HPS are associated with specific liver epigenome changes, which are specially related to hepatic innervation, neuro-hormonal signaling, apoptotic processes, gut bacterial translocation and vascular remodeling. These findings point to the notion that future functional research on the role of methylation changes in the liver is needed to further address unanswered aspect. This may help to shed further light in the pathophysiological mechanisms underlying HPS and better guide its current frustrating therapy.

## Supporting information

S1 File (DOCX)

S1 Fig (A) Network dendrogram of unsupervised hierarchical clustering of the co-methylation network. In the cluster each line represents a probe, which corresponds to a single gene. Probes under the same branch are co-methylated (top). Modules correspond to branches of the dendrogram and were assigned colors for visualization (bottom). (B) Number of single genes contained in each module.
(TIF)

S2 Fig (A)-(E) Scatterplots between module membership measure (X-axis) and the gene significance for DLco of the five selected modules: brown, blue, cyan, darkgrey and salmon.
(TIF)
