# Supplementary material for: Liver epigenome changes in patients with hepatopulmonary syndrome: A pilot study
Source: PLoS One. 2021 Feb 25;16(2):e0245046. doi: 10.1371/journal.pone.0245046 (PMC7906328; doi:10.1371/journal.pone.0245046)
Supplement: S1 File — (DOCX) [file pone.0245046.s001.docx]

S1 File

February 19, 21

ON-LINE SUPPLEMENT

**LIVER EPIGENOME CHANGES IN PATIENTS WITH HEPATOPULMONARY SYNDROME: A PILOT STUDY**

Núria Mendoza^1,2^, Eva Rivas^3,4^, Roberto Rodriguez-Roisin^1,2,5,6^, Tamara Garcia^1^, Miquel Bruguera^2,5,6^, Àlvar Agustí ^1,2,5,6^, Rosa Faner^1,2^*.

**On line supplement:** **Tables**: 5

**S1 Table.** **Genes with positive and negative FC ordered by |logFC| (logFC >|0.4|; p value <0.0009).**

| **Positive FC** | | | | **Negative FC** | | | |
| --- | --- | --- | --- | --- | --- | --- | --- |
| **Gene** | **Probe ID** | **logFC** | **P.Value** | **Gene** | **Probe ID** | **logFC** | **P.Value** |
| **MBIP** | cg26865109 | -1.34 | 0.000510724 | **PDE1C** | cg04494298 | 1.40 | 0.000739715 |
| **SLC22A23** | cg01397117 | -1.30 | 0.000273222 | **LOC200772** | cg24682458 | 1.21 | 0.000540184 |
| **RAP1GDS1** | cg18936471 | -1.21 | 0.000351401 | **MAML2** | cg24022532 | 1.19 | 0.00072106 |
| **ORMDL3** | cg09155575 | -1.18 | 6.47E-05 | **4 GBP** | cg00273257 | 1.17 | 1.50E-05 |
| **SH3RF3** | cg17889815 | -1.18 | 0.000586624 | **ZNF436-AS1** | cg27058404 | 1.16 | 0.000510384 |
| **OXR1** | cg06180882 | -1.15 | 0.000844001 | **ARID5B** | cg10682219 | 1.16 | 0.000769856 |
| **SULT1C3** | cg14612684 | -1.13 | 0.000116483 | **WDR89** | cg10006408 | 1.14 | 0.000510388 |
| **GARNL3** | cg22188408 | -1.12 | 0.000452334 | **FER** | cg15225701 | 1.13 | 0.000129419 |
| **EXOC6B** | cg14819352 | -1.07 | 0.000179488 | **HIVEP2** | cg05786041 | 1.12 | 8.13E-05 |
| **SPDL1** | cg21588667 | -1.03 | 0.000272984 | **FOXK1** | cg14582057 | 1.12 | 0.000361024 |
| **FAM133B** | cg16189569 | -1.02 | 0.000285709 | **FAM168A** | cg16322681 | 1.10 | 1.62E-05 |
| **RFXAP** | cg03968783 | -1.02 | 0.000638683 | **ARHGEF10** | cg15414698 | 1.10 | 0.000135146 |
| **TCTN1** | cg18863210 | -1.01 | 0.000183702 | **LHX4** | cg16999099 | 1.10 | 0.000554541 |
| **VCL** | cg16467231 | -1.01 | 0.000878484 | **MGC70857** | cg00633473 | 1.10 | 0.000729688 |
| **ZNF684** | cg05040778 | -1.00 | 0.000462034 | **ANKRD28** | cg22424370 | 1.09 | 8.14E-05 |
| **FUT10** | cg06570872 | -0.99 | 0.000145606 | **HKR1** | cg05280698 | 1.07 | 0.000127 |
| **DDX60** | cg06648848 | -0.98 | 0.000318824 | **SCAMP3** | cg24034289 | 1.06 | 9.95E-05 |
| **NRG4** | cg00371829 | -0.98 | 0.000385064 | **CCND3** | cg12151296 | 1.06 | 0.0002776 |
| **TRIP13** | cg16646689 | -0.98 | 0.000602098 | **ALDH3B1** | cg14871333 | 1.06 | 0.000380402 |
| **VAV3** | cg19103456 | -0.97 | 2.44E-05 | **SPEN** | cg08892464 | 1.05 | 6.32E-05 |
| **MITF** | cg06640206 | -0.97 | 0.000225158 | **HK1** | cg19170991 | 1.02 | 0.000162802 |
| **SPATA18** | cg22852065 | -0.97 | 0.00051962 | **ADRA2A** | cg26926521 | 1.02 | 0.000272345 |
| **NR5A1** | cg16666160 | -0.96 | 0.000290801 | **UGP2** | cg19915305 | 1.02 | 0.000646397 |
| **HLA-DRB5** | cg15708909 | -0.96 | 0.000542109 | **VTI1A** | cg00592484 | 1.01 | 5.20E-05 |
| **FH** | cg13811454 | -0.95 | 0.000567597 | **XPO1** | cg26131881 | 0.97 | 7.14E-06 |
| **LINC00309** | cg21621819 | -0.94 | 0.000527721 | **BCO2** | cg02168558 | 0.97 | 0.000170037 |
| **MYT1** | cg11839596 | -0.93 | 4.74E-05 | **DIP2C** | cg19462116 | 0.97 | 0.000398002 |
| **CNTNAP2** | cg16508202 | -0.92 | 7.92E-06 | **ARHGAP27** | cg02200722 | 0.97 | 0.000517109 |
| **PAX8** | cg23156509 | -0.91 | 5.56E-05 | **TM4SF1** | cg23379566 | 0.97 | 0.000605977 |
| **ASCC1** | cg06054726 | -0.91 | 0.000702913 | **RCHY1** | cg13431998 | 0.96 | 7.63E-05 |
| **TMCC1** | cg01630688 | -0.90 | 0.000393913 | **IL20RA** | cg10474136 | 0.95 | 0.000401715 |
| **TNPO1** | cg06624956 | -0.90 | 0.000422896 | **KIAA0652** | cg20541870 | 0.95 | 0.000421543 |
| **TSNARE1** | cg00084202 | -0.90 | 0.000597136 | **TRIL** | cg22348673 | 0.95 | 0.000741205 |
| **ATP13A5** | cg05503824 | -0.89 | 0.000385679 | **ZNF467** | cg23749760 | 0.94 | 0.000438506 |
| **GALNT9** | cg07151572 | -0.89 | 0.000478902 | **HACL1** | cg13483696 | 0.94 | 0.00063085 |
| **MAML3** | cg08390519 | -0.89 | 0.000511775 | **IL15RA** | cg09290866 | 0.92 | 0.000518275 |
| **UBR5** | cg03701868 | -0.88 | 0.00020272 | **MFHAS1** | cg21712946 | 0.92 | 0.000705312 |
| **C3orf22** | cg10014862 | -0.88 | 0.000297423 | **NAALAD2** | cg05500015 | 0.91 | 0.000208764 |
| **COQ10B** | cg05059169 | -0.88 | 0.000450776 | **CD177** | cg05275595 | 0.91 | 0.000309277 |
| **CPNE1** | cg15917394 | -0.87 | 9.19E-05 | **TCN2** | cg00788739 | 0.91 | 0.00065489 |
| **OR11G2** | cg13183731 | -0.87 | 0.000455782 | **CCNB1IP1** | cg00129453 | 0.89 | 0.000393727 |
| **EXOC2** | cg01737284 | -0.86 | 0.000336127 | **PUF60** | cg19191888 | 0.89 | 0.000489152 |
| **SERPINB12** | cg00005215 | -0.84 | 0.0002105 | **TMEM132D** | cg22386460 | 0.88 | 5.15E-05 |
| **TENM2** | cg26758826 | -0.84 | 0.000222712 | **MAP2K3** | cg18881501 | 0.88 | 0.000495088 |
| **SH3BP2** | cg05435398 | -0.84 | 0.000513456 | **SERPINB6** | cg19042435 | 0.87 | 0.000495942 |
| **HEXB** | cg14491530 | -0.84 | 0.0008012 | **CCDC129** | cg15049533 | 0.87 | 0.000727017 |
| **RNLS** | cg16503585 | -0.82 | 0.000278562 | **DCAF5** | cg16121759 | 0.84 | 5.34E-05 |
| **PTPRB** | cg05455252 | -0.82 | 0.000562676 | **UBA3** | cg21635584 | 0.84 | 0.00017114 |
| **ADAMTS2** | cg26429655 | -0.81 | 0.000231596 | **EXD3** | cg06323727 | 0.84 | 0.000182845 |
| **MAP9** | cg16382392 | -0.81 | 0.000820144 | **CYFIP1** | cg16650050 | 0.84 | 0.000189413 |
| **TBC1D2B** | cg07690703 | -0.80 | 8.34E-05 | **EDNRB** | cg13434989 | 0.84 | 0.000632654 |
| **AIRE** | cg03146797 | -0.80 | 0.000183535 | **ABCC8** | cg13185308 | 0.83 | 0.000188265 |
| **C14orf39** | cg02394003 | -0.80 | 0.000382295 | **ITPRIP** | cg13559810 | 0.83 | 0.000451496 |
| **RUFY2** | cg08667362 | -0.80 | 0.000858194 | **DNAJC12** | cg19810771 | 0.83 | 0.000530961 |
| **C10orf142** | cg19100384 | -0.79 | 4.44E-05 | **PAX2** | cg07214490 | 0.83 | 0.000779699 |
| **CELSR1** | cg11982890 | -0.79 | 0.000289177 | **BNC2** | cg06893785 | 0.82 | 0.000271591 |
| **ARL6IP5** | cg21575951 | -0.79 | 0.000409652 | **GSTCD** | cg02005549 | 0.82 | 0.000774313 |
| **PIK3AP1** | cg20318778 | -0.79 | 0.000507616 | **CMIP** | cg00123622 | 0.82 | 0.000849285 |
| **ARHGAP40** | cg11964389 | -0.78 | 0.000135954 | **ALPL** | cg14781605 | 0.81 | 0.000204908 |
| **ISLR** | cg11335960 | -0.78 | 0.000161152 | **LOC101928937** | cg25537060 | 0.81 | 0.000222053 |
| **MTUS1** | cg00277065 | -0.78 | 0.000252652 | **OSTC** | cg15184315 | 0.81 | 0.00075186 |
| **ESRRG** | cg01833485 | -0.78 | 0.000469575 | **2 DKK** | cg00594011 | 0.80 | 0.000313558 |
| **WEE2** | cg12976456 | -0.78 | 0.000542025 | **TEP1** | cg13442001 | 0.80 | 0.000370737 |
| **RNF146** | cg09178900 | -0.78 | 0.000764847 | **PROX1** | cg23084895 | 0.80 | 0.00040819 |
| **PEX14** | cg24294013 | -0.77 | 0.000135927 | **MEF2C** | cg26650655 | 0.80 | 0.000466571 |
| **KLHDC4** | cg00246208 | -0.77 | 0.000868895 | **HYI** | cg03548730 | 0.80 | 0.000476921 |
| **C3orf59** | cg10590657 | -0.77 | 0.000884857 | **CHST4** | cg25494045 | 0.80 | 0.000837077 |
| **RPL29P2** | cg14421980 | -0.76 | 0.000121683 | **LOXL2** | cg02183676 | 0.80 | 0.000871764 |
| **JMJD1C** | cg19299499 | -0.76 | 0.000187732 | **TCEA3** | cg18167160 | 0.79 | 1.72E-05 |
| **ZMAT4** | cg00330581 | -0.76 | 0.000350636 | **ZYG11A** | cg20287790 | 0.79 | 0.000257487 |
| **ACVR1** | cg10328244 | -0.76 | 0.000402271 | **RAP1B** | cg22510091 | 0.79 | 0.000748582 |
| **IL15** | cg25690285 | -0.75 | 4.88E-05 | **C18orf8** | cg01374372 | 0.79 | 0.000833333 |
| **CRAMP1L** | cg15231222 | -0.75 | 5.09E-05 | **MIR519C** | cg15930678 | 0.78 | 0.000240284 |
| **CDH22** | cg24101578 | -0.75 | 0.000138309 | **IGFBP7** | cg15696506 | 0.78 | 0.000697535 |
| **TUBB** | cg09510976 | -0.75 | 0.000447013 | **RAD18** | cg13041470 | 0.78 | 0.000724396 |
| **OPCML** | cg24249286 | -0.75 | 0.000536583 | **ID4** | cg17252960 | 0.78 | 0.000811749 |
| **FGFR3** | cg09576124 | -0.75 | 0.000652432 | **EGLN1** | cg03079863 | 0.77 | 0.000232077 |
| **LOC100288162** | cg07324498 | -0.75 | 0.000805117 | **FNTB** | cg19637116 | 0.77 | 0.000622229 |
| **ZFHX4** | cg19776984 | -0.75 | 0.000883823 | **ZBTB45** | cg06638023 | 0.77 | 0.000624889 |
| **RMND5B** | cg17406092 | -0.74 | 0.000110168 | **KRAS** | cg18249781 | 0.76 | 9.34E-05 |
| **ZNF592** | cg12958080 | -0.74 | 0.000131579 | **SLC39A8** | cg23846145 | 0.76 | 0.000335161 |
| **GALK2** | cg10156207 | -0.74 | 0.000223277 | **CCDC149** | cg27605638 | 0.76 | 0.000694736 |
| **LOC389333** | cg05449136 | -0.74 | 0.000543664 | **ANO10** | cg19647107 | 0.75 | 7.41E-05 |
| **GRID1** | cg22688030 | -0.74 | 0.000638643 | **GABRG2** | cg09220361 | 0.75 | 0.000271923 |
| **PRKCA** | cg17160506 | -0.74 | 0.000673032 | **ARHGAP15** | cg11921583 | 0.75 | 0.000382927 |
| **GUCY2EP** | cg16855265 | -0.73 | 8.74E-05 | **SV2B** | cg03940484 | 0.75 | 0.000556618 |
| **KLC1** | cg20781954 | -0.73 | 0.000190382 | **CDC37L1** | cg01296653 | 0.75 | 0.00056512 |
| **CACNA1H** | cg06521960 | -0.73 | 0.000232263 | **MFSD7** | cg17952262 | 0.75 | 0.000708614 |
| **ZRANB2-AS2** | cg25392814 | -0.73 | 0.000344268 | **WSCD2** | cg24018665 | 0.74 | 0.000128446 |
| **ACE** | cg02440279 | -0.73 | 0.000447179 | **DUSP10** | cg18282005 | 0.74 | 0.00019359 |
| **ZNF397** | cg13882921 | -0.73 | 0.000840963 | **FNDC5** | cg20951444 | 0.74 | 0.000360689 |
| **STK32B** | cg21637176 | -0.72 | 0.000238108 | **C17orf51** | cg14995803 | 0.73 | 0.000201391 |
| **MAGI2** | cg01239112 | -0.72 | 0.000321314 | **HOGA1** | cg05979855 | 0.73 | 0.000275018 |
| **MICALL1** | cg03660208 | -0.72 | 0.000426598 | **TMEM68** | cg03274117 | 0.73 | 0.000365197 |
| **LOC101928107** | cg16435573 | -0.72 | 0.000498648 | **ZNF778** | cg26489188 | 0.73 | 0.000501403 |
| **KRT20** | cg23901700 | -0.72 | 0.00058576 | **AMIGO1** | cg21692308 | 0.72 | 0.000472159 |
| **LRRC1** | cg14858446 | -0.72 | 0.000670333 | **NUDT13** | cg16758988 | 0.72 | 0.000507645 |
| **MAP2** | cg09533013 | -0.72 | 0.000836095 | **SFRS18** | cg08676600 | 0.71 | 0.00030031 |
| **VWCE** | cg22189307 | -0.72 | 0.000845079 | **NR2E1** | cg25383479 | 0.71 | 0.00034631 |
| **MYO18A** | cg02376256 | -0.71 | 4.31E-05 | **PRR3** | cg13413789 | 0.71 | 0.000441569 |
| **ADAMTS16** | cg05666479 | -0.71 | 0.000122482 | **NDUFA10** | cg05622382 | 0.71 | 0.000670575 |
| **NAALADL2** | cg15672986 | -0.71 | 0.000228391 | **MIR200C** | cg16642299 | 0.71 | 0.000717754 |
| **IMMP2L** | cg13508208 | -0.71 | 0.000300488 | **GPR97** | cg01932823 | 0.71 | 0.000810807 |
| **CATSPERB** | cg04689773 | -0.71 | 0.000372553 | **ULK1** | cg23088672 | 0.70 | 0.000128963 |
| **SHROOM3** | cg10026495 | -0.71 | 0.000400252 | **LHFPL2** | cg06759890 | 0.70 | 0.000241928 |
| **GLG1** | cg17730623 | -0.71 | 0.000440403 | **LOC100130238** | cg08788842 | 0.70 | 0.000329347 |
| **SIRT1** | cg13796676 | -0.71 | 0.000570024 | **TRIM15** | cg21248305 | 0.70 | 0.000332216 |
| **INA** | cg23003534 | -0.71 | 0.000585808 | **HIST1H3C** | cg10199913 | 0.70 | 0.000430163 |
| **ABCA7** | cg16482616 | -0.71 | 0.000767726 | **BRCA1** | cg12182452 | 0.70 | 0.00061387 |
| **LMO7** | cg17356355 | -0.70 | 0.000163561 | **SLC36A4** | cg12518360 | 0.70 | 0.000686283 |
| **UBE2U** | cg23068989 | -0.70 | 0.000237414 | **LOC100126784** | cg07189401 | 0.70 | 0.000718996 |
| **ASCL1** | cg27420520 | -0.70 | 0.000331827 | **HIST1H2BB** | cg02221866 | 0.70 | 0.000860825 |
| **ARRDC5** | cg20230308 | -0.70 | 0.000437381 | **PACSIN2** | cg16321057 | 0.70 | 0.000866471 |
| **CDH2** | cg24776465 | -0.70 | 0.000563878 | **SH3PXD2B** | cg08378342 | 0.69 | 0.000118 |
| **STX8** | cg11797480 | -0.69 | 0.000202553 | **OCEL1** | cg04746699 | 0.69 | 0.000333919 |
| **CFAP44** | cg03827687 | -0.69 | 0.00023556 | **RBKS** | cg08648550 | 0.69 | 0.000703688 |
| **GPC5** | cg02082760 | -0.69 | 0.000407045 | **CLSTN1** | cg23616100 | 0.69 | 0.000734598 |
| **SPEG** | cg07582167 | -0.69 | 0.000552473 | **DUOXA2** | cg10042437 | 0.69 | 0.000782767 |
| **FBXW7** | cg07517746 | -0.69 | 0.000564755 | **RELN** | cg17923358 | 0.68 | 0.000164335 |
| **LINC00673** | cg04667945 | -0.69 | 0.000578268 | **STK32C** | cg07400503 | 0.68 | 0.0002421 |
| **HAUS6** | cg07898631 | -0.69 | 0.000620535 | **EPHA6** | cg11301768 | 0.68 | 0.000276246 |
| **SPINLW1** | cg10379687 | -0.69 | 0.000649622 | **TTLL10** | cg14000174 | 0.68 | 0.000345057 |
| **CD81** | cg26382697 | -0.69 | 0.000674687 | **DLG2** | cg02707033 | 0.68 | 0.000548573 |
| **GSK3B** | cg07440417 | -0.69 | 0.000714338 | **NAP1L5** | cg12759554 | 0.68 | 0.000660065 |
| **CEMIP** | cg24762504 | -0.69 | 0.000719627 | **TMEM184A** | cg03564557 | 0.68 | 0.000783106 |
| **MYT1L** | cg03322328 | -0.69 | 0.000827447 | **LINC00620** | cg04907198 | 0.67 | 0.000364089 |
| **ADAP1** | cg04007890 | -0.68 | 0.00018558 | **ZNF99** | cg20314184 | 0.67 | 0.000387296 |
| **BANP** | cg05802204 | -0.68 | 0.000205777 | **H2AFY** | cg23782426 | 0.67 | 0.000432212 |
| **DOCK2** | cg23598132 | -0.68 | 0.000276417 | **C3orf31** | cg06184463 | 0.67 | 0.000867324 |
| **RAD51B** | cg27368135 | -0.68 | 0.000495744 | **APOBEC3B** | cg23225498 | 0.67 | 0.000899881 |
| **NTN1** | cg17316435 | -0.68 | 0.000575724 | **SNX3** | cg23763053 | 0.66 | 0.000120 |
| **FA2H** | cg22133237 | -0.68 | 0.000642452 | **FOXRED2** | cg22463596 | 0.66 | 0.000128605 |
| **RPS6KA2** | cg09340511 | -0.68 | 0.000647023 | **TRPV6** | cg23449659 | 0.66 | 0.000219321 |
| **C17orf47** | cg11890622 | -0.67 | 0.000124415 | **LOC100129066** | cg13466725 | 0.66 | 0.000304382 |
| **CYP11A1** | cg23660703 | -0.67 | 0.000185019 | **AKR1B15** | cg17342491 | 0.66 | 0.000434569 |
| **C21orf34** | cg12166520 | -0.67 | 0.000297225 | **LYRM7** | cg26355399 | 0.66 | 0.00062618 |
| **LRTOMT** | cg27225973 | -0.67 | 0.00039301 | **ATP13A2** | cg17434062 | 0.65 | 0.000822909 |
| **LOC101929563** | cg09560061 | -0.67 | 0.000500178 | **PDE7B** | cg27306443 | 0.64 | 0.000308976 |
| **SOGA1** | cg20376545 | -0.67 | 0.000670138 | **BRSK2** | cg01660630 | 0.64 | 0.000394486 |
| **NXN** | cg08965655 | -0.67 | 0.000708821 | **MIR130A** | cg16520038 | 0.64 | 0.000498125 |
| **PRIMA1** | cg24160740 | -0.67 | 0.000798933 | **KCNH4** | cg25634207 | 0.64 | 0.000543349 |
| **DENND1B** | cg07479119 | -0.67 | 0.000822915 | **RXFP1** | cg09599228 | 0.64 | 0.000674441 |
| **PTPRS** | cg02463653 | -0.67 | 0.000835269 | **FRMD4B** | cg08872313 | 0.64 | 0.000841669 |
| **RNF2** | cg13274427 | -0.67 | 0.000841226 | **LAMA2** | cg10671433 | 0.63 | 6.68E-05 |
| **ANKRD11** | cg04200026 | -0.66 | 9.89E-05 | **UPP1** | cg07247721 | 0.63 | 0.00010049 |
| **TAS1R2** | cg11806635 | -0.66 | 0.000119361 | **CDH6** | cg04132555 | 0.63 | 0.000462718 |
| **CCDC12** | cg16113216 | -0.66 | 0.000122031 | **SPTBN4** | cg05764628 | 0.63 | 0.000664776 |
| **NADSYN1** | cg12474705 | -0.66 | 0.000221083 | **ESCO2** | cg06556616 | 0.63 | 0.000668089 |
| **TBCD** | cg18879998 | -0.66 | 0.000305057 | **C19orf81** | cg07116872 | 0.63 | 0.000755426 |
| **CORO7-PAM16** | cg07187342 | -0.66 | 0.000391088 | **TOP1MT** | cg11809417 | 0.62 | 0.000163226 |
| **SNAP91** | cg21704750 | -0.66 | 0.000495406 | **SCUBE1** | cg26536894 | 0.62 | 0.000360853 |
| **ANO1** | cg04620917 | -0.66 | 0.000866031 | **SZT2** | cg10361120 | 0.62 | 0.000518975 |
| **SARM1** | cg08044516 | -0.65 | 0.000174185 | **CCDC85C** | cg15650039 | 0.61 | 0.000415333 |
| **AVIL** | cg11993266 | -0.65 | 0.000586846 | **MAP4K4** | cg21550141 | 0.60 | 0.000228812 |
| **BRF1** | cg24037673 | -0.65 | 0.000690336 | **KALRN** | cg05056583 | 0.60 | 0.000333912 |
| **ANGPT2** | cg05052368 | -0.65 | 0.000707581 | **LOC100289511** | cg25112611 | 0.60 | 0.000350385 |
| **CUX1** | cg01716016 | -0.65 | 0.000748943 | **COPB2** | cg18313110 | 0.60 | 0.00044766 |
| **TTC39A** | cg24797045 | -0.64 | 3.46E-05 | **DYNC1I1** | cg11180624 | 0.60 | 0.000595465 |
| **INPP5A** | cg07326267 | -0.64 | 0.000100734 | **SARS2** | cg24148629 | 0.59 | 0.000135366 |
| **PDGFRB** | cg04176448 | -0.64 | 0.000136988 | **NMNAT2** | cg10856032 | 0.59 | 0.000188464 |
| **HDAC4** | cg05704850 | -0.64 | 0.000160937 | **TSSC1** | cg13109482 | 0.58 | 0.000384663 |
| **LAMA5** | cg07447854 | -0.64 | 0.000427161 | **PTPRN2** | cg20764780 | 0.57 | 0.00032713 |
| **CNOT6** | cg09544279 | -0.64 | 0.000431828 | **TNXB** | cg04210056 | 0.57 | 0.000561219 |
| **ARHGAP24** | cg25157032 | -0.64 | 0.000454814 | **ALKBH3** | cg26099549 | 0.57 | 0.000598567 |
| **CSF1R** | cg03921345 | -0.64 | 0.000703738 | **MIR4435-2HG** | cg15778059 | 0.57 | 0.000837282 |
| **HCG9** | cg01260810 | -0.63 | 0.000117511 | **LNX1** | cg11807829 | 0.56 | 0.000344354 |
| **DDX11L1** | cg14817997 | -0.63 | 0.00034638 | **LNPEP** | cg07248814 | 0.55 | 0.000225783 |
| **LINC01607** | cg01265861 | -0.63 | 0.000482777 | **CALN1** | cg03229590 | 0.55 | 0.000299439 |
| **SND1** | cg00092551 | -0.63 | 0.00059976 | **NEAT1** | cg09411730 | 0.55 | 0.000368509 |
| **SHANK2** | cg05337228 | -0.63 | 0.000660005 | **RUFY3** | cg06109873 | 0.55 | 0.00077685 |
| **ZNF662** | cg07448333 | -0.63 | 0.00082548 | **EXOC7** | cg13149032 | 0.55 | 0.000838667 |
| **DGKG** | cg17685523 | -0.63 | 0.000848264 | **RNASEK** | cg26657400 | 0.54 | 0.000504897 |
| **GLOD4** | cg06569993 | -0.62 | 0.000128778 | **MYH11** | cg08789739 | 0.54 | 0.000610492 |
| **GALNT14** | cg19849093 | -0.62 | 0.000305376 | **HDGF** | cg04875476 | 0.54 | 0.000789243 |
| **GRM6** | cg00301483 | -0.61 | 0.000182783 | **OSBP2** | cg06696028 | 0.54 | 0.00085822 |
| **BMPR2** | cg21911195 | -0.61 | 0.000250656 | **CBFA2T3** | cg05953373 | 0.53 | 0.000510199 |
| **LINC01258** | cg05397248 | -0.61 | 0.00029762 | **PHLDB1** | cg11736167 | 0.53 | 0.000847902 |
| **HKDC1** | cg05470908 | -0.60 | 0.000161378 | **IPO9** | cg00326651 | 0.52 | 0.000448759 |
| **CALML3-AS1** | cg10241132 | -0.60 | 0.000276019 | **PCBP1** | cg09175843 | 0.51 | 0.000390196 |
| **ANKRD33B** | cg15908114 | -0.60 | 0.000341858 | **VPS52** | cg00058113 | 0.51 | 0.000481693 |
| **TMEM131** | cg14079020 | -0.60 | 0.000483222 | **BCRP2** | cg22970157 | 0.51 | 0.000634057 |
| **PCNX** | cg21463350 | -0.60 | 0.000655839 | **CASC20** | cg24716369 | 0.50 | 0.000316403 |
| **CLDN10** | cg12160467 | -0.60 | 0.000804282 | **PREX1** | cg17602683 | 0.50 | 0.000356841 |
| **VPS16** | cg15533884 | -0.59 | 0.000198171 | **NUP210** | cg22748554 | 0.50 | 0.000499499 |
| **TP53I11** | cg23489437 | -0.59 | 0.000589109 | **KCNA4** | cg15310492 | 0.49 | 0.000397598 |
| **ZMYM6** | cg17938303 | -0.59 | 0.000776116 | **PLXND1** | cg07078269 | 0.48 | 0.000576302 |
| **ANKMY1** | cg27000612 | -0.59 | 0.000793977 | **SH2B2** | cg27482432 | 0.47 | 0.000754155 |
| **DEAF1** | cg06861788 | -0.59 | 0.000826467 | **BEGAIN** | cg03515464 | 0.46 | 0.000629286 |
| **C1orf201** | cg03140978 | -0.58 | 0.000268148 | **ST7L** | cg17861791 | 0.45 | 0.000495336 |
| **MIR29B1** | cg02000032 | -0.58 | 0.000300298 | **CNN3** | cg18344652 | 0.41 | 0.000858722 |
| **SAMD11** | cg00582671 | -0.58 | 0.000342631 |  |  |  |  |
| **RD3** | cg06753273 | -0.58 | 0.000345285 |  |  |  |  |
| **F11** | cg09786229 | -0.58 | 0.00047907 |  |  |  |  |
| **LAMC3** | cg09265360 | -0.57 | 0.000303268 |  |  |  |  |
| **HDAC9** | cg12210898 | -0.57 | 0.000314828 |  |  |  |  |
| **PLEC** | cg08763272 | -0.57 | 0.000329304 |  |  |  |  |
| **PRDM16** | cg22389615 | -0.57 | 0.000444095 |  |  |  |  |
| **UNC13A** | cg02617655 | -0.57 | 0.000660959 |  |  |  |  |
| **MIR4688** | cg03832078 | -0.57 | 0.000782794 |  |  |  |  |
| **FLJ31306** | cg17323045 | -0.57 | 0.000867616 |  |  |  |  |
| **HSPB8** | cg09819940 | -0.56 | 0.00015678 |  |  |  |  |
| **MSH2** | cg15552882 | -0.56 | 0.000317668 |  |  |  |  |
| **PRICKLE2** | cg08356738 | -0.56 | 0.000439478 |  |  |  |  |
| **PIEZO1** | cg22741612 | -0.56 | 0.000440319 |  |  |  |  |
| **PEX26** | cg13499896 | -0.56 | 0.000478225 |  |  |  |  |
| **KIR2DL4** | cg03247919 | -0.56 | 0.000487588 |  |  |  |  |
| **SCD5** | cg07844828 | -0.56 | 0.000758885 |  |  |  |  |
| **BLK** | cg07764073 | -0.56 | 0.000792915 |  |  |  |  |
| **TSC2** | cg06243965 | -0.56 | 0.00080288 |  |  |  |  |
| **AATK** | cg20887396 | -0.56 | 0.000814937 |  |  |  |  |
| **MUC2** | cg24631065 | -0.55 | 0.00014754 |  |  |  |  |
| **BSND** | cg00307818 | -0.55 | 0.000297749 |  |  |  |  |
| **CASP7** | cg07787708 | -0.55 | 0.000573032 |  |  |  |  |
| **PPM1D** | cg20136726 | -0.55 | 0.000702254 |  |  |  |  |
| **LMAN1L** | cg05262938 | -0.55 | 0.000767684 |  |  |  |  |
| **C8orf42** | cg00066748 | -0.55 | 0.000769671 |  |  |  |  |
| **PUS10** | cg24211619 | -0.54 | 0.000170985 |  |  |  |  |
| **RASAL2** | cg25003878 | -0.54 | 0.000227909 |  |  |  |  |
| **BLOC1S1** | cg21054231 | -0.54 | 0.000387533 |  |  |  |  |
| **PER1** | cg27073561 | -0.53 | 0.000199653 |  |  |  |  |
| **OLA1** | cg09579094 | -0.53 | 0.000208223 |  |  |  |  |
| **C2orf81** | cg06800942 | -0.53 | 0.000357461 |  |  |  |  |
| **APOH** | cg19253201 | -0.53 | 0.000488394 |  |  |  |  |
| **TRIM22** | cg17025642 | -0.53 | 0.000651008 |  |  |  |  |
| **SLC12A7** | cg23110957 | -0.52 | 0.000391999 |  |  |  |  |
| **DNAH17** | cg09296416 | -0.52 | 0.000483283 |  |  |  |  |
| **ZNF783** | cg19614632 | -0.52 | 0.000542281 |  |  |  |  |
| **MIR2052HG** | cg01405527 | -0.52 | 0.00078401 |  |  |  |  |
| **PAPL** | cg08062233 | -0.52 | 0.000829639 |  |  |  |  |
| **AGTPBP1** | cg13024416 | -0.51 | 0.000291526 |  |  |  |  |
| **GPR132** | cg16486653 | -0.51 | 0.000668328 |  |  |  |  |
| **ORC4** | cg23686623 | -0.51 | 0.000772262 |  |  |  |  |
| **MACROD2** | cg19826779 | -0.50 | 0.000567648 |  |  |  |  |
| **JMJD7-PLA2G4B** | cg25852492 | -0.50 | 0.000649301 |  |  |  |  |
| **LOC100507194** | cg15497960 | -0.50 | 0.000707338 |  |  |  |  |
| **FGD2** | cg09832916 | -0.49 | 0.000545349 |  |  |  |  |
| **BPI** | cg12959196 | -0.49 | 0.000705934 |  |  |  |  |
| **LOC349196** | cg16275722 | -0.48 | 0.000341986 |  |  |  |  |
| **ZNF19** | cg00888605 | -0.47 | 0.000408976 |  |  |  |  |
| **TRRAP** | cg12813151 | -0.47 | 0.000566156 |  |  |  |  |
| **RHOBTB1** | cg11883238 | -0.47 | 0.000620358 |  |  |  |  |
| **KCNC1** | cg16404865 | -0.47 | 0.000626433 |  |  |  |  |
| **ODZ2** | cg26874611 | -0.47 | 0.000719693 |  |  |  |  |
| **MICAL3** | cg02481739 | -0.46 | 0.000564264 |  |  |  |  |
| **LOC102724663** | cg26321842 | -0.46 | 0.00078179 |  |  |  |  |
| **RIMS1** | cg14878736 | -0.46 | 0.000868303 |  |  |  |  |
| **SLC4A2** | cg19502841 | -0.46 | 0.000889017 |  |  |  |  |
| **FCHSD2** | cg09611499 | -0.45 | 0.000897375 |  |  |  |  |
| **DAND5** | cg15415411 | -0.44 | 0.000616046 |  |  |  |  |
| **SLC16A8** | cg00111823 | -0.44 | 0.000633022 |  |  |  |  |
| **FUS** | cg12513648 | -0.43 | 0.00072499 |  |  |  |  |
| **GUSBL2** | cg25281020 | -0.40 | 0.000832125 |  |  |  |  |
| **PLCD1** | cg10718914 | -0.40 | 0.000852382 |  |  |  |  |

**S2 Table.** **Histopathology characterization of the study population.**

| **Variable** | **With HPS** (n=4) | **Without HPS** (n=4) |
| --- | --- | --- |
| **Type of cirrhosis** |  |  |
| Micronodular  Macronodular  Mixed  Incomplete septal cirrhosis | 2  1  1  0 | 1  0  2  1 |
| **Inflammation** |  |  |
| Absence  Mild  Severe | 0  2  2 | 4  0  0 |
| **Vascular proliferation within fibrous septa** |  |  |
| Yes  No | 3  1 | 4  0 |
| **Sinusoidal dilatation** |  |  |
| Yes  No | 2  2 | 2  2 |
| **Parenchymal necrosis areas** | 2  2 | 0  4 |
| Yes  No |  |  |
| **Colangiolar proliferation** | 0  1  3 | 1  2  1 |
| Absence  Mild  Extensive |  |  |

**S3 Table. Gene Ontology enrichment in the general list filtered by p value 0.0009 (FDR<0.05).**

| **Gene Ontology description and :: ID** | **FDR** **p value** | **Genes** |
| --- | --- | --- |
| neuron projection morphogenesis::GO:0048812 | 0.000184 | AMIGO1,BMPR2, BRSK2, CDH2, CNTNAP2 |
| plasma membrane bounded cell projection morphogenesis::GO:0120039 | 0.000184 | AMIGO1, BMPR2, BRSK2, CDH2,  CNTNAP2 |
| generation of neurons::GO:0048699 | 0.000184 | ABCC8,AGTPBP1, AMIGO1, ASCL1, AVIL |
| cell morphogenesis involved in neuron differentiation::GO:0048667 | 0.000184 | AMIGO1, BMPR2, BRSK2, CDH2,  CSF1R |
| regulation of neuron differentiation::GO:0045664 | 0.0005203 | AMIGO1, ASCL1, AVIL, BMPR2,  BRSK2 |
| neuron projection development::GO:0031175 | 0.0005203 | AMIGO1, AVIL, BLOC1S1, BMPR2, BRSK2 |
| regulation of neuron projection development::GO:0010975 | 0.0005203 | AMIGO1, AVIL, BMPR2, BRSK2, CDH2 |
| axonogenesis::GO:0007409 | 0.001418 | AMIGO1, BMPR2, BRSK2, CDH2, CSF1R |
| regulation of neurogenesis::GO:0050767 | 0.001418 | ABCC8, AMIGO1, ASCL1, AVIL,  BMPR2 |
| regulation of plasma membrane bounded cell projection organization::GO:0120035 | 0.001418 | ADAMTS16, AMIGO1, AVIL, BMPR2, BRSK2 |
| regulation of cell morphogenesis involved in differentiation::GO:0010769 | 0.002795 | AMIGO1, BMPR2, BRSK2, CDH2, CUX1 |
| regulation of synaptic vesicle clustering::GO:2000807 | 0.002917 | BRSK2, CDH2, MAGI2, TEP1 |
| positive regulation of neuron differentiation::GO:0045666 | 0.002917 | AMIGO1, ASCL1, AVIL, BMPR2, CPNE1 |
| axon development::GO:0061564 | 0.004199 | AMIGO1, BMPR2, BRSK2, CDH2, CSF1R |
| postsynaptic density organization::GO:0097106 | 0.004241 | CDH2, DLG2, TEP1, PTPRS, RELN |
| phosphorylation::GO:0016310 | 0.01626 | AATK, ABCA7, ACE, ACVR1, ADRA2A |
| peptidyl-amino acid modification::GO:0018193 | 0.01676 | ACE, ACVR1, ADRA2A, AGTPBP1, ATP13A2 |
| central nervous system neuron differentiation::GO:0021953 | 0.01725 | AGTPBP1, ASCL1, CSF1R, ID4, LHX4 |
| synaptic vesicle clustering::GO:0097091 | 0.01866 | BRSK2, CDH2, MAGI2, TEP1 |
| regulation of axonogenesis::GO:0050770 | 0.02001 | AMIGO1, BMPR2, BRSK2, CDH2, CYFIP1 |
| gliogenesis::GO:0042063 | 0.02001 | ABCC8, ARHGEF10, ASCL1, CDH2, DUSP10 |
| regulation of brown fat cell differentiation::GO:0090335 | 0.02064 | DUSP10, FNDC5, PRDM16, SIRT1 |
| positive regulation of neuron projection development::GO:0010976 | 0.02064 | AMIGO1, AVIL, BMPR2, CUX1, CYFIP1 |
| peptidyl-lysine deacetylation::GO:0034983 | 0.02256 | HDAC4, HDAC9, SIRT1 |
| cellular protein modification process::GO:0006464 | 0.02259 | AATK, ABCA7, ACE, ACVR1, ADRA2A |
| positive regulation of GTPase activity::GO:0043547 | 0.02439 | ADAP1, ARHGAP15, ARHGAP24, ARHGAP27, ARHGAP40 |
| regulation of cytoskeleton organization::GO:0051493 | 0.02439 | ARHGAP40, ARHGEF10, AVIL, BRCA1, CELSR1 |
| protein phosphorylation::GO:0006468 | 0.02439 | AATK, ABCA7, ACE, ACVR1, ADRA2A |
| regulation of GTPase activity::GO:0043087 | 0.02439 | ADAP1, ARHGAP15, ARHGAP24, ARHGAP27, ARHGAP40 |
| axon extension::GO:0048675 | 0.02439 | BMPR2, CYFIP1, GSK3B, MAP2, NTN1 |
| pronephric field specification::GO:0039003 | 0.02439 | PAX2, PAX8 |
| negative regulation of mesenchymal cell apoptotic process involved in metanephric nephron morphogenesis::GO:0072305 | 0.02439 | PAX2, PAX8 |
| regulation of mesenchymal cell apoptotic process involved in metanephric nephron morphogenesis::GO:0072304 | 0.02439 | PAX2, PAX8 |
| mesenchymal stem cell maintenance involved in metanephric nephron morphogenesis::GO:0072309 | 0.02439 | PAX2, PAX8 |
| mesenchymal cell apoptotic process involved in metanephric nephron morphogenesis::GO:1901147 | 0.02439 | PAX2, PAX8 |
| central nervous system neuron axonogenesis::GO:0021955 | 0.02439 | NR2E1, TEP1, SPTBN4, SZT2, TCTN1 |
| central nervous system neuron development::GO:0021954 | 0.03243 | ASCL1, MAP2, NR2E1, TEP1, SPTBN4 |
| endothelial cell migration::GO:0043542 | 0.03618 | ANGPT2, APOH, BMPR2, FBXW7, HDAC9 |
| histone H3 deacetylation::GO:0070932 | 0.03645 | HDAC4, HDAC9, PER1, SIRT1 |
| positive regulation of hydrolase activity::GO:0051345 | 0.03935 | ADAP1, APOH, ARHGAP15, ARHGAP24, ARHGAP27 |
| chemical synaptic transmission::GO:0007268 | 0.03967 | CDH2, CLSTN1, CYFIP1, DLG2, EGLN1 |
| anterograde trans-synaptic signaling::GO:0098916 | 0.03967 | CDH2, CLSTN1, CYFIP1, DLG2, EGLN1 |
| negative regulation of activin receptor signaling pathway::GO:0032926 | 0.04139 | ACVR1, DAND5, MAGI2 |
| brown fat cell differentiation::GO:0050873 | 0.04966 | DUSP10, FNDC5, PRDM16, SH2B2, SIRT1 |

**S4 Table. Gene Ontology enrichment in positive and negative FC lists (FDR<0.05).**

| **Gene Ontology description and :: ID** | **FDR** **p value** | **Genes** |
| --- | --- | --- |
| **Positive FC** | | |
| peptidyl-lysine deacetylation:: GO:0034983 | 0.04486 | HDAC4, HDAC9, SIRT1 |
| histone H3 deacetylation::GO:0070932 | 0.04486 | HDAC4, HDAC9, PER1, SIRT1 |
| positive regulation of hydrolase activity:: GO:0051345 | 0.04486 | ADAP1, APOH, ARHGAP24, ARHGAP40, ARL6IP5 |
| **Negative FC** | | |
| cell morphogenesis involved in neuron differentiation:: GO:0048667 | 0.01084 | AMIGO1,BRSK2,CYFIP1, KALRN, LAMA2 |
| axonogenesis:: GO:0007409 | 0.01084 | AMIGO1,BRSK2,CYFIP1, LAMA2, LHX4 |
| regulation of neurogenesis:: GO: 0050767 | 0.01084 | ABCC8, AMIGO1, BRSK2, CYFIP1, DUSP10 |
| neuron projection morphogenesis::GO:0048812 | 0.01304 | AMIGO1,BRSK2,CYFIP1, KALRN, LAMA2 |
| axon development::GO:0061564 | 0.01304 | AMIGO1,BRSK2,CYFIP1, LAMA2, LHX4 |
| regulation of neuron differentiation::GO:0045664 | 0.01304 | AMIGO1,BRSK2,CYFIP1, EDNRB, ID4 |
| plasma membrane bounded cell projection morphogenesis:: GO:0120039 | 0.01304 | AMIGO1,BRSK2,CYFIP1, KALRN, LAMA2 |
| gliogenesis::GO:0042063 | 0.01304 | ABCC8, ARHGEF10, DUSP10, ID4, KRAS |
| central nervous system axonogenesis::GO:0021955 | 0.01997 | NR2E1, TEP1, SPTBN4, SZT2 |
| regulation of cell morphogenesis involved in differentiation:: GO:0010769 | 0.01997 | AMIGO1,BRSK2,CYFIP1, KALRN, NR2E1 |
| regulation of neuron projection development:: GO:0010975 | 0.01997 | AMIGO1,BRSK2,CYFIP1, KALRN, MAP4K4 |
| chemical synaptic transmission::GO:0007268 | 0.01997 | CLSTN1, CYFIP1, DLG2, EGLN1, GABRG2 |
| anterograde trans-synaptic signaling::GO:0098916 | 0.01997 | CLSTN1, CYFIP1, DLG2, EGLN1, GABRG2 |
| neuron projection development::GO:0031175 | 0.02679 | AMIGO1,BRSK2,CYFIP1, KALRN, LAMA2 |
| negative regulation of gliogenesis::GO:0014014 | 0.03018 | ABCC8, DUSP10, ID4, NR2E1 |
| generation of neurons:: GO:0048699 | 0.03018 | ABCC8, AMIGO1, BRSK2, CYFIP1, DUSP10 |
| negative regulation of histone H3-K4 methylation::GO:0051572 | 0.03018 | BRCA1, H2AFY |
| central nervous system neuron differentiation::GO:0021953 | 0.05072 | ID4, LHX4, NR2E1, PROX1, TEP1 |

**S5 Table. Gene Ontology enrichment in Brown, Blue and Darkgrey modules (FDR<0.05).**

| **Gene Ontology Description and: ID** | **# genes** | **p value** | **FDR p value** |
| --- | --- | --- | --- |
| **Brown Module** | | | |
| regulation of cellular macromolecule biosynthetic process::GO:2000112 | 360 | 6.37E-08 | 0.000266 |
| transcription, DNA-templated::GO:0006351 | 338 | 2.94E-07 | 0.0006136 |
| nucleic acid-templated transcription::GO:0097659 | 338 | 4.70E-07 | 0.0006548 |
| regulation of transcription, DNA-templated::GO:0006355 | 323 | 7.64E-07 | 0.0006769 |
| regulation of nucleic acid-templated transcription::GO:1903506 | 324 | 9.84E-07 | 0.0006769 |
| RNA biosynthetic process::GO:0032774 | 345 | 1.07E-06 | 0.0006769 |
| regulation of RNA biosynthetic process::GO:2001141 | 325 | 1.13E-06 | 0.0006769 |
| regulation of protein serine/threonine kinase activity::GO:0071900 | 61 | 2.71E-06 | 0.001417 |
| protein phosphorylation::GO:0006468 | 181 | 3.80E-06 | 0.001764 |
| regulation of kinase activity::GO:0043549 | 88 | 7.44E-06 | 0.002931 |
| regulation of protein kinase activity::GO:0045859 | 83 | 7.72E-06 | 0.002931 |
| apoptotic process::GO:0006915 | 174 | 9.54E-06 | 0.003319 |
| cellular response to lipid::GO:0071396 | 62 | 1.11E-05 | 0.003549 |
| regulation of protein phosphorylation::GO:0001932 | 131 | 1.24E-05 | 0.003696 |
| regulation of apoptotic process::GO:0042981 | 139 | 1.35E-05 | 0.003755 |
| regulation of MAPK cascade::GO:0043408 | 77 | 1.58E-05 | 0.004118 |
| branching involved in salivary gland morphogenesis::GO:0060445 | 9 | 1.70E-05 | 0.004172 |
| cellular response to organic cyclic compound::GO:0071407 | 62 | 1.83E-05 | 0.004245 |
| regulation of phosphorylation::GO:0042325 | 137 | 2.57E-05 | 0.005363 |
| cardiac muscle cell differentiation::GO:0055007 | 19 | 2.63E-05 | 0.005363 |
| protein localization to organelle::GO:0033365 | 96 | 2.70E-05 | 0.005363 |
| phosphorylation::GO:0016310 | 204 | 3.73E-05 | 0.00648 |
| regulation of phosphate metabolic process::GO:0019220 | 155 | 3.78E-05 | 0.00648 |
| positive regulation of kinase activity::GO:0033674 | 60 | 3.83E-05 | 0.00648 |
| regulation of protein modification process::GO:0031399 | 159 | 3.88E-05 | 0.00648 |
| regulation of ERK1 and ERK2 cascade::GO:0070372 | 35 | 4.22E-05 | 0.006778 |
| regulation of MAP kinase activity::GO:0043405 | 43 | 5.22E-05 | 0.008082 |
| positive regulation of protein kinase activity::GO:0045860 | 56 | 7.56E-05 | 0.01128 |
| chordate embryonic development::GO:0043009 | 65 | 8.10E-05 | 0.01166 |
| embryo development ending in birth or egg hatching::GO:0009792 | 65 | 1.00E-04 | 0.01393 |
| striated muscle tissue development::GO:0014706 | 43 | 0.0001151 | 0.01399 |
| negative regulation of protein serine/threonine kinase activity::GO:0071901 | 21 | 0.0001161 | 0.01399 |
| positive regulation of protein modification process::GO:0031401 | 113 | 0.0001178 | 0.01399 |
| smooth muscle cell differentiation::GO:0051145 | 12 | 0.0001178 | 0.01399 |
| negative regulation of MAPK cascade::GO:0043409 | 24 | 0.0001247 | 0.01399 |
| positive regulation of transferase activity::GO:0051347 | 70 | 0.0001284 | 0.01399 |
| transcription from RNA polymerase II promoter::GO:0006366 | 185 | 0.0001301 | 0.01399 |
| negative regulation of apoptotic process::GO:0043066 | 85 | 0.0001358 | 0.01399 |
| ERK1 and ERK2 cascade::GO:0070371 | 35 | 0.0001372 | 0.01399 |
| positive regulation of protein serine/threonine kinase activity::GO:0071902 | 39 | 0.0001385 | 0.01399 |
| epithelial cell migration::GO:0010631 | 32 | 0.0001405 | 0.01399 |
| epithelial cell differentiation::GO:0030855 | 63 | 0.0001431 | 0.01399 |
| cellular response to steroid hormone stimulus::GO:0071383 | 33 | 0.000144 | 0.01399 |
| regulation of transcription from RNA polymerase II promoter::GO:0006357 | 169 | 0.0001513 | 0.01436 |
| cellular response to growth factor stimulus::GO:0071363 | 67 | 0.0001661 | 0.01541 |
| negative regulation of cellular macromolecule biosynthetic process::GO:2000113 | 127 | 0.0001709 | 0.01552 |
| enzyme linked receptor protein signaling pathway::GO:0007167 | 98 | 0.0001746 | 0.01552 |
| regulation of cardiac muscle cell differentiation::GO:2000725 | 8 | 0.0001889 | 0.01644 |
| negative regulation of cell size::GO:0045792 | 5 | 0.0001934 | 0.01649 |
| positive regulation of proteolysis::GO:0045862 | 44 | 0.0002192 | 0.01831 |
| transmembrane receptor protein tyrosine kinase signaling pathway::GO:0007169 | 72 | 0.000233 | 0.01867 |
| phosphatidylinositol-mediated signaling::GO:0048015 | 29 | 0.000234 | 0.01867 |
| regulation of establishment of protein localization to mitochondrion::GO:1903747 | 21 | 0.0002369 | 0.01867 |
| negative regulation of transcription, DNA-templated::GO:0045892 | 110 | 0.0002606 | 0.02016 |
| inositol lipid-mediated signaling::GO:0048017 | 29 | 0.0003029 | 0.02257 |
| activation of protein kinase activity::GO:0032147 | 38 | 0.0003058 | 0.02257 |
| positive regulation of phosphate metabolic process::GO:0045937 | 103 | 0.000308 | 0.02257 |
| cellular response to unfolded protein::GO:0034620 | 21 | 0.0003312 | 0.02267 |
| negative regulation of cardiac muscle tissue development::GO:0055026 | 7 | 0.0003414 | 0.02267 |
| positive regulation of transcription, DNA-templated::GO:0045893 | 127 | 0.000345 | 0.02267 |
| positive regulation of nucleic acid-templated transcription::GO:1903508 | 127 | 0.000345 | 0.02267 |
| positive regulation of phosphorylation::GO:0042327 | 93 | 0.0003489 | 0.02267 |
| positive regulation of apoptotic process::GO:0043065 | 62 | 0.0003525 | 0.02267 |
| cellular response to bacterial lipopeptide::GO:0071221 | 4 | 0.0003631 | 0.02267 |
| response to bacterial lipopeptide::GO:0070339 | 4 | 0.0003631 | 0.02267 |
| cellular response to bacterial lipoprotein::GO:0071220 | 4 | 0.0003631 | 0.02267 |
| regulation of proteolysis::GO:0030162 | 74 | 0.0003678 | 0.02267 |
| negative regulation of nucleic acid-templated transcription::GO:1903507 | 113 | 0.000369 | 0.02267 |
| negative regulation of protein phosphorylation::GO:0001933 | 46 | 0.0003787 | 0.02292 |
| glucocorticoid receptor signaling pathway::GO:0042921 | 6 | 0.0004008 | 0.02392 |
| negative regulation of RNA biosynthetic process::GO:1902679 | 114 | 0.0004212 | 0.02478 |
| positive regulation of RNA biosynthetic process::GO:1902680 | 128 | 0.0004528 | 0.02627 |
| phosphatidylinositol 3-kinase signaling::GO:0014065 | 24 | 0.0004651 | 0.02648 |
| regulation of cardiac muscle cell contraction::GO:0086004 | 8 | 0.0004691 | 0.02648 |
| positive regulation of protein phosphorylation::GO:0001934 | 89 | 0.0004831 | 0.02672 |
| negative regulation of MAP kinase activity::GO:0043407 | 14 | 0.0004861 | 0.02672 |
| regulation of G1/S transition of mitotic cell cycle::GO:2000045 | 21 | 0.0005068 | 0.02749 |
| negative regulation of transcription from RNA polymerase II promoter::GO:0000122 | 77 | 0.0005484 | 0.029 |
| regulation of cell cycle G1/S phase transition::GO:1902806 | 22 | 0.0005484 | 0.029 |
| NAD biosynthetic process::GO:0009435 | 6 | 0.0006022 | 0.03119 |
| activation of MAPK activity::GO:0000187 | 22 | 0.0006048 | 0.03119 |
| in utero embryonic development::GO:0001701 | 39 | 0.000654 | 0.03233 |
| establishment of protein localization to mitochondrion::GO:0072655 | 26 | 0.0006543 | 0.03233 |
| positive regulation of cellular protein catabolic process::GO:1903364 | 26 | 0.0006543 | 0.03233 |
| negative regulation of steroid biosynthetic process::GO:0010894 | 7 | 0.0006607 | 0.03233 |
| salivary gland morphogenesis::GO:0007435 | 9 | 0.0006656 | 0.03233 |
| regulation of mitotic cell cycle phase transition::GO:1901990 | 37 | 0.0006941 | 0.03333 |
| positive regulation of mitochondrial membrane permeability involved in apoptotic process::GO:1902110 | 11 | 0.000735 | 0.0345 |
| mitochondrial outer membrane permeabilization involved in programmed cell death::GO:1902686 | 11 | 0.000735 | 0.0345 |
| bud elongation involved in lung branching::GO:0060449 | 4 | 0.0007985 | 0.03665 |
| response to bacterial lipoprotein::GO:0032493 | 4 | 0.0007985 | 0.03665 |
| protein localization to mitochondrion::GO:0070585 | 26 | 0.0008389 | 0.03809 |
| regulation of stress-activated MAPK cascade::GO:0032872 | 27 | 0.0008487 | 0.03812 |
| corticosteroid receptor signaling pathway::GO:0031958 | 6 | 0.0008741 | 0.0385 |
| positive regulation of MAPK cascade::GO:0043410 | 53 | 0.0008816 | 0.0385 |
| endothelium development::GO:0003158 | 18 | 0.0008849 | 0.0385 |
| embryonic skeletal system morphogenesis::GO:0048704 | 16 | 0.0009666 | 0.04162 |
| positive regulation of proteolysis involved in cellular protein catabolic process::GO:1903052 | 24 | 0.001036 | 0.0439 |
| positive regulation of mitochondrial membrane permeability::GO:0035794 | 11 | 0.001041 | 0.0439 |
| regulation of cardiac muscle tissue development::GO:0055024 | 12 | 0.001057 | 0.04417 |
| carboxylic acid metabolic process::GO:0019752 | 86 | 0.001077 | 0.04454 |
| heart contraction::GO:0060047 | 32 | 0.00109 | 0.04464 |
| platelet-derived growth factor receptor signaling pathway::GO:0048008 | 10 | 0.001168 | 0.04736 |
| regulation of phosphatidylinositol 3-kinase signaling::GO:0014066 | 21 | 0.001219 | 0.04896 |
| embryonic digestive tract morphogenesis::GO:0048557 | 6 | 0.001231 | 0.04899 |
| regulation of muscle hypertrophy::GO:0014743 | 9 | 0.001263 | 0.04976 |
| response to dexamethasone::GO:0071548 | 8 | 0.001286 | 0.05022 |
| positive regulation of MAP kinase activity::GO:0043406 | 28 | 0.001335 | 0.05163 |
| activation of phospholipase A2 activity::GO:0032431 | 3 | 0.001429 | 0.05401 |
| negative regulation of interleukin-1-mediated signaling pathway::GO:2000660 | 3 | 0.001429 | 0.05401 |
| positive regulation of DNA replication::GO:0045740 | 11 | 0.001444 | 0.05401 |
| stress-activated MAPK cascade::GO:0051403 | 31 | 0.001457 | 0.05401 |
| regulation of muscle contraction::GO:0006937 | 21 | 0.001461 | 0.05401 |
| **Blue Module** | | | |
| cellular protein modification process::GO:0006464 | 871 | 2,21E-11 | 1,28E-07 |
| generation of neurons::GO:0048699 | 331 | 1,59E-06 | 4.60E-06 |
| neuron differentiation::GO:0030182 | 302 | 4.86E-09 | 7.91E-06 |
| neurogenesis::GO:0022008 | 347 | 5,48E-06 | 7.91E-06 |
| phosphorylation::GO:0016310 | 511 | 8,94E-06 | 1,03E-02 |
| neuron projection morphogenesis::GO:0048812 | 153 | 2,82E-05 | 2,72E-02 |
| protein phosphorylation::GO:0006468 | 432 | 4,84E-05 | 3,52E-02 |
| cell morphogenesis involved in neuron differentiation::GO:0048667 | 142 | 4.88E-08 | 3,52E-02 |
| regulation of neurogenesis::GO:0050767 | 179 | 1,33E-04 | 8,51E-02 |
| regulation of GTPase activity::GO:0043087 | 182 | 3,80E-04 | 0.0002197 |
| positive regulation of GTPase activity::GO:0043547 | 168 | 8,37E-04 | 0.0004396 |
| positive regulation of hydrolase activity::GO:0051345 | 228 | 1,04E-03 | 0.0005021 |
| regulation of neuron projection development::GO:0010975 | 114 | 1,36E-03 | 0.0006053 |
| regulation of axon extension involved in axon guidance::GO:0048841 | 19 | 1,75E-03 | 0.0006692 |
| regulation of neuron differentiation::GO:0045664 | 147 | 1.83E-06 | 0.0006692 |
| axon extension involved in axon guidance::GO:0048846 | 21 | 1.97E-06 | 0.0006692 |
| neuron projection extension involved in neuron projection guidance::GO:1902284 | 21 | 1.97E-06 | 0.0006692 |
| calcium ion transport::GO:0006816 | 105 | 2,16E-03 | 0.0006921 |
| axon guidance::GO:0007411 | 69 | 3,08E-03 | 0.000935 |
| regulation of calcium ion transport::GO:0051924 | 63 | 4,46E-03 | 0.001287 |
| metal ion transport::GO:0030001 | 199 | 4,70E-03 | 0.001294 |
| dephosphorylation::GO:0016311 | 119 | 6,53E-03 | 0.001714 |
| divalent metal ion transport::GO:0070838 | 114 | 7,19E-03 | 0.001805 |
| axon development::GO:0061564 | 120 | 8,89E-03 | 0.002139 |
| divalent inorganic cation transport::GO:0072511 | 114 | 1,03E-02 | 0.002383 |
| regulation of protein modification process::GO:0031399 | 373 | 1,40E-02 | 0.003105 |
| axonogenesis::GO:0007409 | 111 | 1,49E-02 | 0.003192 |
| regulation of phosphate metabolic process::GO:0019220 | 362 | 1,79E-02 | 0.003662 |
| camera-type eye development::GO:0043010 | 83 | 1,84E-02 | 0.003662 |
| regulation of metal ion transport::GO:0010959 | 88 | 2,04E-02 | 0.003921 |
| negative regulation of axon extension involved in axon guidance::GO:0048843 | 14 | 2.14E-05 | 0.003987 |
| cilium morphogenesis::GO:0060271 | 72 | 2,97E-02 | 0.005365 |
| cation transport::GO:0006812 | 227 | 3,68E-02 | 0.006437 |
| chemical synaptic transmission::GO:0007268 | 150 | 4,04E-02 | 0.006486 |
| anterograde trans-synaptic signaling::GO:0098916 | 150 | 4,04E-02 | 0.006486 |
| trans-synaptic signaling::GO:0099537 | 150 | 4,04E-02 | 0.006486 |
| protein dephosphorylation::GO:0006470 | 76 | 4,70E-02 | 0.007331 |
| regulation of transcription from RNA polymerase II promoter::GO:0006357 | 402 | 5,71E-02 | 0.008555 |
| nerve growth factor signaling pathway::GO:0038180 | 7 | 5,82E-02 | 0.008555 |
| enzyme linked receptor protein signaling pathway::GO:0007167 | 226 | 5,92E-02 | 0.008555 |
| regulation of cell morphogenesis involved in differentiation::GO:0010769 | 74 | 6,65E-02 | 0.009365 |
| G1/S transition of mitotic cell cycle::GO:0000082 | 67 | 7,23E-02 | 0.009888 |
| protein localization to plasma membrane::GO:0072659 | 60 | 7,45E-02 | 0.009888 |
| ion transmembrane transport::GO:0034220 | 224 | 7,53E-02 | 0.009888 |
| protein localization to cell periphery::GO:1990778 | 63 | 8,09E-02 | 0.01038 |
| small GTPase mediated signal transduction::GO:0007264 | 142 | 0.0001137 | 0.01427 |
| regulation of ion transmembrane transport::GO:0034765 | 101 | 0.0001428 | 0.01754 |
| photoreceptor cell development::GO:0042461 | 18 | 0.0001468 | 0.01758 |
| transcription from RNA polymerase II promoter::GO:0006366 | 437 | 0.0001492 | 0.01758 |
| cellular response to organic cyclic compound::GO:0071407 | 127 | 0.0001556 | 0.01798 |
| transmembrane receptor protein tyrosine kinase signaling pathway::GO:0007169 | 161 | 0.0001629 | 0.0183 |
| neuroepithelial cell differentiation::GO:0060563 | 20 | 0.0001648 | 0.0183 |
| establishment of protein localization to plasma membrane::GO:0090002 | 43 | 0.0002236 | 0.02437 |
| cellular hypotonic response::GO:0071476 | 6 | 0.0002704 | 0.02892 |
| sphingolipid biosynthetic process::GO:0030148 | 33 | 0.0003056 | 0.03209 |
| regulation of dephosphorylation::GO:0035303 | 48 | 0.0003484 | 0.03593 |
| regulation of cell cycle G1/S phase transition::GO:1902806 | 44 | 0.0003754 | 0.03761 |
| regulation of phosphorylation::GO:0042325 | 304 | 0.0003837 | 0.03761 |
| cation transmembrane transport::GO:0098655 | 165 | 0.0003842 | 0.03761 |
| regulation of small GTPase mediated signal transduction::GO:0051056 | 77 | 0.0003937 | 0.0379 |
| semaphorin-plexin signaling pathway::GO:0071526 | 16 | 0.0004049 | 0.03834 |
| positive regulation of protein modification process::GO:0031401 | 254 | 0.000422 | 0.03931 |
| Ras protein signal transduction::GO:0007265 | 82 | 0.0004882 | 0.04476 |
| regulation of cation transmembrane transport::GO:1904062 | 58 | 0.000534 | 0.0482 |
| regulation of G1/S transition of mitotic cell cycle::GO:2000045 | 41 | 0.0005525 | 0.04909 |
| regulation of protein phosphorylation::GO:0001932 | 284 | 0.000566 | 0.04953 |
| positive regulation of RNA biosynthetic process::GO:1902680 | 298 | 0.0005862 | 0.05053 |
| neural crest cell migration::GO:0001755 | 20 | 0.0006019 | 0.05113 |
| stress-activated MAPK cascade::GO:0051403 | 67 | 0.0006154 | 0.05151 |
| calcium ion transmembrane transport::GO:0070588 | 70 | 0.0006823 | 0.05513 |
| regulation of calcium ion transmembrane transport::GO:1903169 | 36 | 0.0006944 | 0.05513 |
| positive regulation of transcription, DNA-templated::GO:0045893 | 293 | 0.0006968 | 0.05513 |
| positive regulation of nucleic acid-templated transcription::GO:1903508 | 293 | 0.0006968 | 0.05513 |
| regulation of MAPK cascade::GO:0043408 | 157 | 0.0007122 | 0.0552 |
| regulation of cellular macromolecule biosynthetic process::GO:2000112 | 810 | 0.0007167 | 0.0552 |
| **Darkgrey Module** | | | |
| positive regulation of nuclear-transcribed mRNA poly(A) tail shortening::GO:0060213 | 2 | 0.0001084 | 0.01149 |
| regulation of nuclear-transcribed mRNA poly(A) tail shortening::GO:0060211 | 2 | 0.0001084 | 0.01149 |
| miRNA mediated inhibition of translation::GO:0035278 | 2 | 0.0001299 | 0.01149 |
| negative regulation of translation, ncRNA-mediated::GO:0040033 | 2 | 0.0001299 | 0.01149 |
| regulation of translation, ncRNA-mediated::GO:0045974 | 2 | 0.0001299 | 0.01149 |
| phosphatidylinositol-mediated signaling::GO:0048015 | 4 | 0.0001735 | 0.01156 |
| inositol lipid-mediated signaling::GO:0048017 | 4 | 0.0001838 | 0.01156 |
| positive regulation of nuclear-transcribed mRNA catabolic process, deadenylation-dependent decay::GO:1900153 | 2 | 0.0002354 | 0.01156 |
| regulation of nuclear-transcribed mRNA catabolic process, deadenylation-dependent decay::GO:1900151 | 2 | 0.0002354 | 0.01156 |
| positive regulation of mRNA catabolic process::GO:0061014 | 2 | 0.000632 | 0.02793 |
| regulation of mRNA catabolic process::GO:0061013 | 2 | 0.0007874 | 0.03164 |
| nuclear-transcribed mRNA poly(A) tail shortening::GO:0000289 | 2 | 0.001083 | 0.03977 |
| Wnt signaling pathway, calcium modulating pathway::GO:0007223 | 2 | 0.001425 | 0.03977 |
| histone H3-K4 demethylation, trimethyl-H3-K4-specific::GO:0034721 | 1 | 0.00144 | 0.03977 |
| regulation of estradiol secretion::GO:2000864 | 1 | 0.00144 | 0.03977 |
| estradiol secretion::GO:0035938 | 1 | 0.00144 | 0.03977 |
| positive regulation of mRNA metabolic process::GO:1903313 | 2 | 0.002151 | 0.05299 |
| activation of MAPKK activity::GO:0000186 | 2 | 0.002425 | 0.05299 |
| gene silencing by miRNA::GO:0035195 | 2 | 0.002815 | 0.05299 |
| histone H3-R2 methylation::GO:0034970 | 1 | 0.002877 | 0.05299 |
| histone H3-R17 methylation::GO:0034971 | 1 | 0.002877 | 0.05299 |
| positive regulation of base-excision repair::GO:1905053 | 1 | 0.002877 | 0.05299 |
| microtubule sliding::GO:0051012 | 1 | 0.002877 | 0.05299 |
| regulation of base-excision repair::GO:1905051 | 1 | 0.002877 | 0.05299 |
