# Supplementary figures and images for: Liver epigenome changes in patients with hepatopulmonary syndrome: A pilot study
Source: PLoS One. 2021 Feb 25;16(2):e0245046. doi: 10.1371/journal.pone.0245046 (PMC7906328; doi:10.1371/journal.pone.0245046)

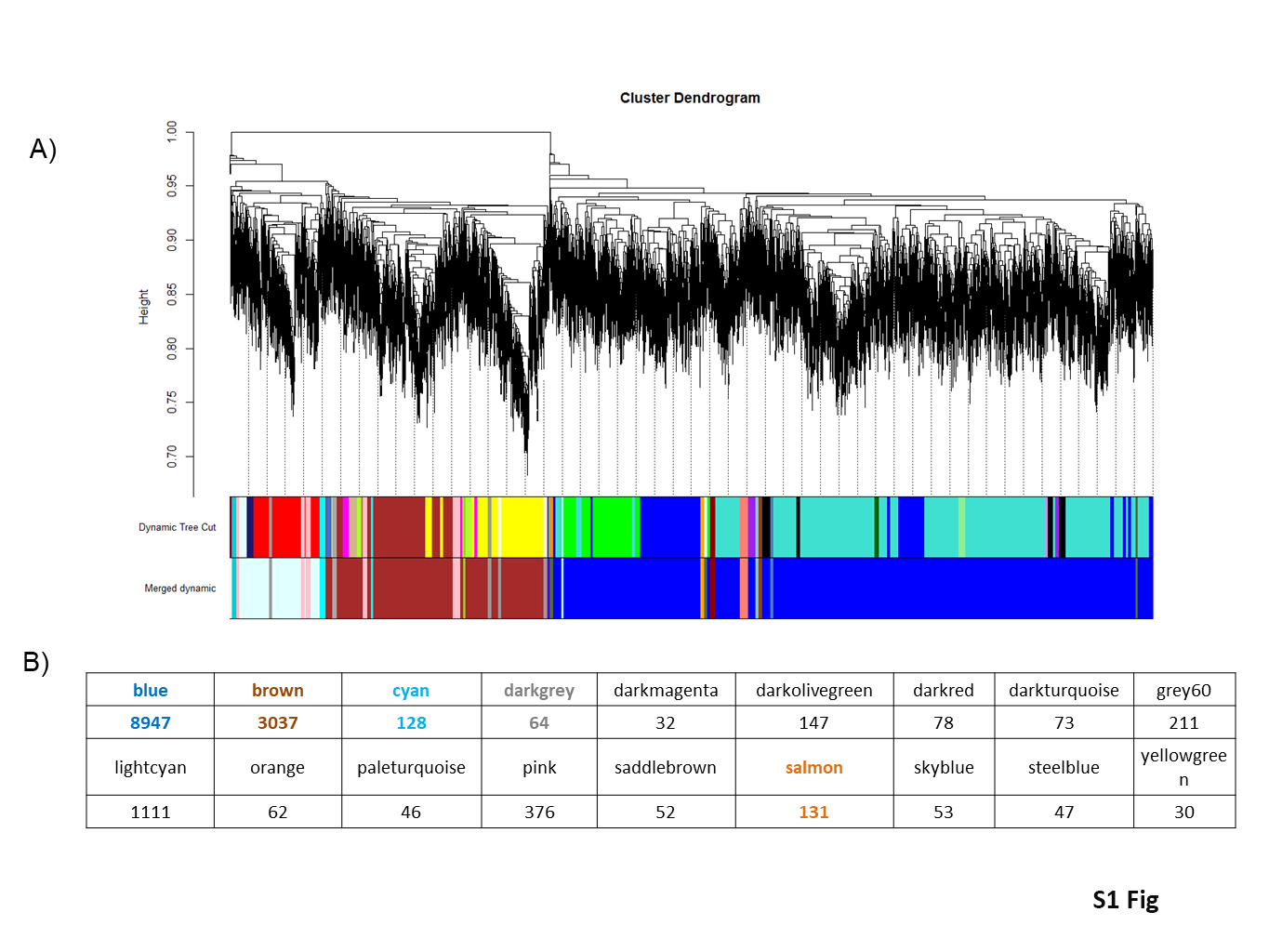

Supplement: S1 Fig — (A) Network dendrogram of unsupervised hierarchical clustering of the co-methylation network. In the cluster each line represents a probe, which corresponds to a single gene. Probes under the same branch are co-methylated (top). Modules correspond to branches of the dendrogram and were assigned colors for visualization (bottom). (B) Number of single genes contained in each module. (TIF) [file pone.0245046.s002.tif]

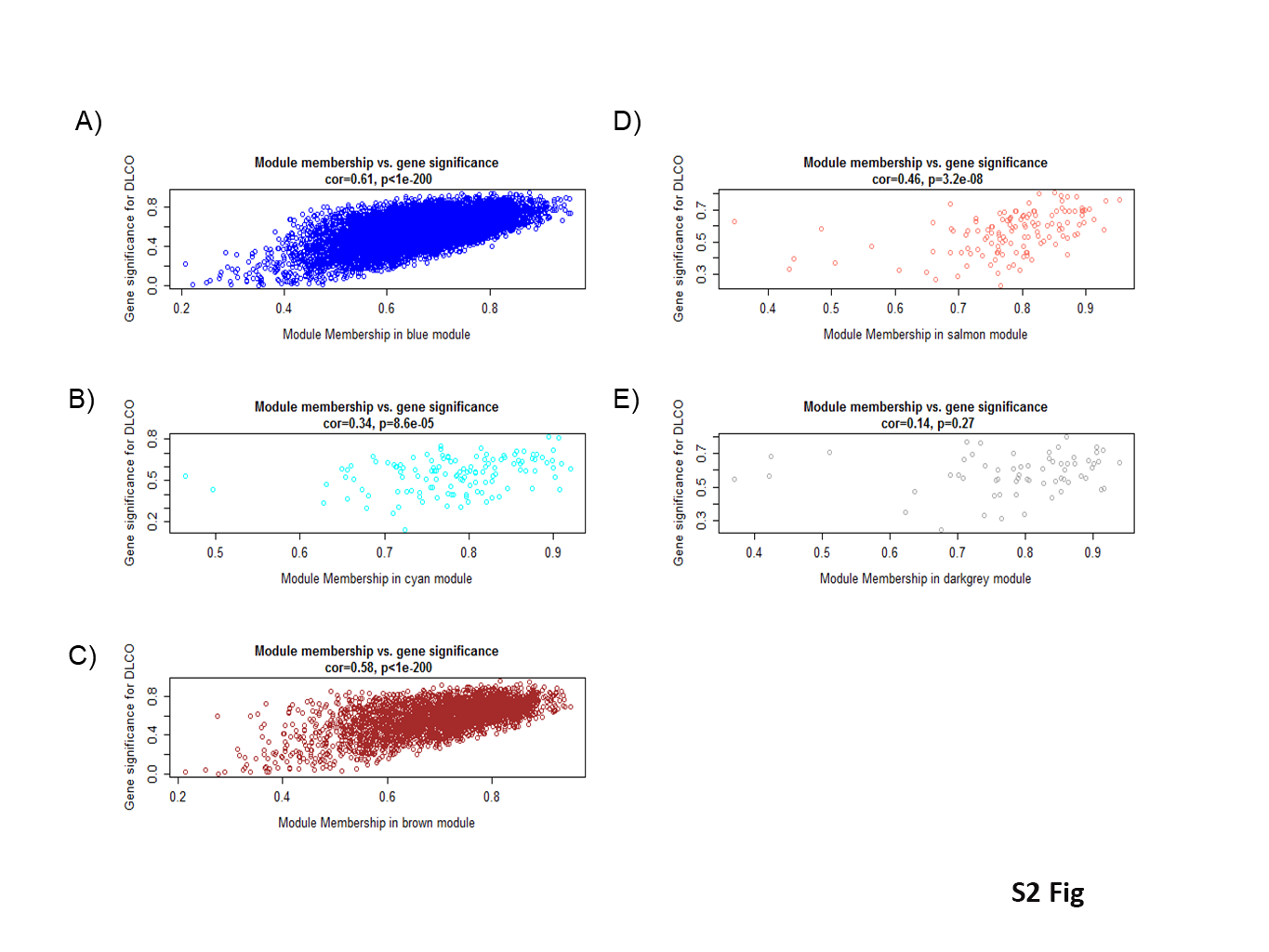

Supplement: S2 Fig — (A)-(E) Scatterplots between module membership measure (X-axis) and the gene significance for DLco of the five selected modules: brown, blue, cyan, darkgrey and salmon. (TIF) [file pone.0245046.s003.tif]
